# Forest stand growth dynamics in Central Europe have accelerated since 1870

**DOI:** 10.1038/ncomms5967

**Published:** 2014-09-12

**Authors:** Hans Pretzsch, Peter Biber, Gerhard Schütze, Enno Uhl, Thomas Rötzer

**Affiliations:** 1 Chair for Forest Growth and Yield Science, Center of Life and Food Sciences Weihenstephan, Technische Universität München, Hans-Carl-von-Carlowitz-Platz 2, 85354 Freising, Germany; 2 Bavarian State Institute of Forestry, Hans-Carl-von-Carlowitz-Platz 1, 85354 Freising, Germany

## Abstract

Forest ecosystems have been exposed to climate change for more than 100 years, whereas the consequences on forest growth remain elusive. Based on the oldest existing experimental forest plots in Central Europe, we show that, currently, the dominant tree species Norway spruce and European beech exhibit significantly faster tree growth (+32 to 77%), stand volume growth (+10 to 30%) and standing stock accumulation (+6 to 7%) than in 1960. Stands still follow similar general allometric rules, but proceed more rapidly through usual trajectories. As forest stands develop faster, tree numbers are currently 17–20% lower than in past same-aged stands. Self-thinning lines remain constant, while growth rates increase indicating the stock of resources have not changed, while growth velocity and turnover have altered. Statistical analyses of the experimental plots, and application of an ecophysiological model, suggest that mainly the rise in temperature and extended growing seasons contribute to increased growth acceleration, particularly on fertile sites.

## Abstract (web summary)

The growth dynamics of forest ecosystems undergoing climatic change are not well understood. Here Pretzsch *et al*. show that two of the dominant tree species of Central Europe have undergone significantly accelerated growth dynamics during the past century.

## Graphical abstract

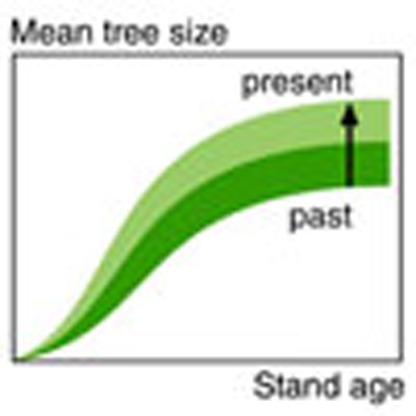

The lifespan of many tree species is over several hundred years; therefore, knowledge regarding tree and forest stand dynamics, and long-term impacts due to environmental change is largely incomplete. Retrospective tree ring analyses can only partly close this knowledge gap, as this method indeed offers insights into tree growth, but not into stand dynamics. Forest inventories primarily assess managed forests, where the influences of climate change and thinning might be coalesced, and difficult to differentiate. Models are frequently used as a means to circumvent data collection and subsequent analyses^1^. However, modelling is no substitute for underlying field data, and the full potential of any modelling approach is only fulfilled when feedback between modelling studies and empirical analyses are achieved. A unique source of information, however, was provided by long-term experimental plots established in approximately 1872, the founding year of the International Union of Forest Research Organisations^2^. These plots, which were surveyed 10–20 times until the present day, provide the longest existing time series data on forest stand dynamics available, approximately 140 years. Originally, the study stands were established to examine stand growth principles^3^, but not for growth trend analysis^2^. In addition to the *per se* uniqueness of the sites, the locations are in Central European regions where the longest time series on driving variables (precipitation and temperature) dates back to 1781. The original data acquisition objective was to support sustainable forestry at a local scale; however, we subsequently used these unique records to quantify and characterize changes in Central European forest growth.

We chose Norway spruce (*Picea abies* (L.) Karst.) and European beech (*Fagus sylvatica* L.) as the study species. These taxa dominate Central Europe’s forests occupying 30%, that is, a total area of 14 × 10^6^ ha of all forest areas. The plots selected for this study represent pure, even-aged stands, which were established by planting or seeding. Site conditions varied broadly, and soils ranged from dry silty sands to moist deep silts. Since the first site observations and records in 1870, the plots were maintained under continuous scientific control, and surveyed on a single tree basis. Therefore, investigators excluded plots and reports impacted by disturbances, including storms or bark beetle infestations. We included only unmanaged, or at most moderately thinned, but always fully stocked plots. This selection resulted in a unique survey data set from 36 spruce and 22 beech plots.

Based on these data we show that both species currently exhibit significantly faster tree growth, stand volume growth and standing stock accumulation than still in 1960 and the decades before. Self-thinning lines remain constant, while growth rates increase indicating the stock of resources have not changed, while growth velocity and turnover have altered. This means stands still follow similar general allometric rules, but proceed more rapidly through usual trajectories. As we can demonstrate, this results in stands currently having lower tree numbers per unit area than past stands at the same age. Our data also reveal that the growth acceleration is stronger on fertile sites, which is supported by scenario runs with an ecophysiological growth model.

## Results

### Changes in stand dynamics and environmental conditions

**Figure 1:**
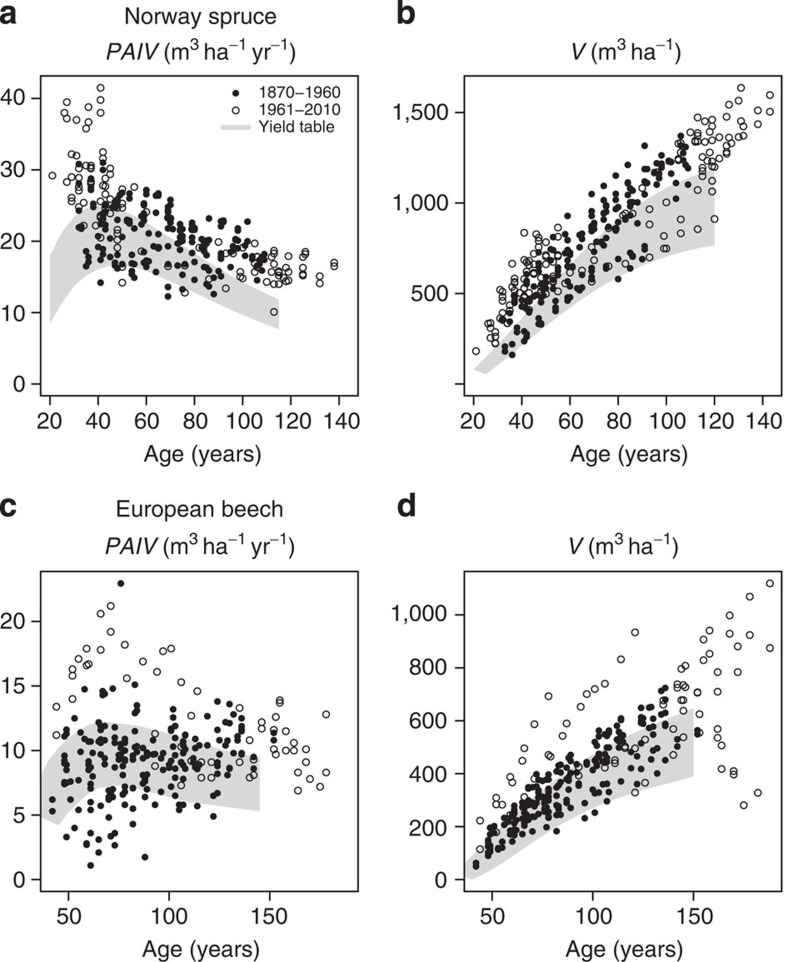
Observed versus expected stand growth for Norway spruce and European beech since 1870. Observed Periodic Annual Volume Increment (*PAIV* in m^3^ ha^−1^ per year) for Norway spruce (**a**) and European beech (**c**), and wood standing stock volume (*V* in m^3^ ha^−1^) for Norway spruce (**b**) and European beech (**d**) up to 1960 (filled symbols) and after 1960 (empty symbols) compared with a common yield table for Norway spruce^4^ (grey section: site index 32–40) and European beech^5^ (grey section: site index I–IV).

First, we pooled our data and compared them to standard yield tables^4^,^5^ (Fig. 1). Yield tables, common forestry tools that tabulate stand growth age dependently, were developed primarily from 1795 to 1965. Yield table data were derived from long-term plot field survey data; and thus they served to represent past growth conditions in this comparison. We found stand growth rates and standing stocks after 1960 (empty symbols in Fig. 1) exceeded the yield table ranges by 50–100%, which called the validity of yield table range data into question, and suggested essential changes in stand dynamics.

**Figure 2:**
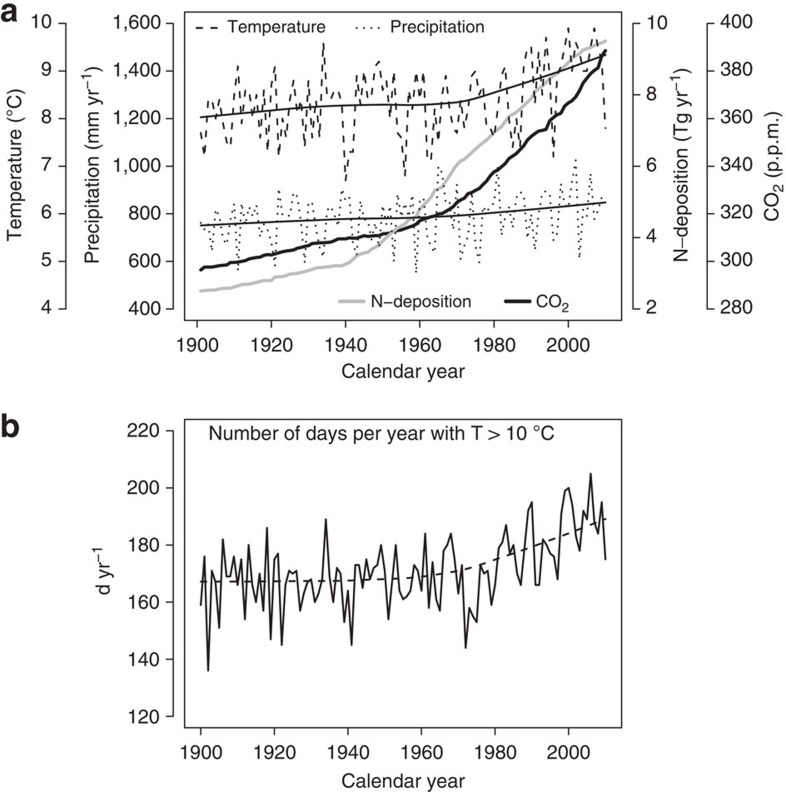
Change in growth conditions for Central Europe since 1900. (**a**) Trend in mean annual air temperature (dashed), annual precipitation (dotted), atmospheric CO_2_-concentration (bold black line) and N-deposition (bold grey). For better trend visualization loess smoothers for temperature and precipitation have been added (thin solid lines). (**b**) Extended annual growing season, expressed by the number of days per year with a mean temperature >10 °C (solid). The dashed line represents a loess smoother. Data sources: Churkina *et al*.^6^, Schönwiese *et al*.^10^

For the same period our plot surveys spanned, we compiled available data on environmental variables reported to drive forest growth dynamics (Fig. 2). For Central Europe, forest environmental and growing conditions exhibited significant changes since the first experimental forest plots were established in 1870 (Fig. 2). During the addressed period, the atmospheric CO_2_ concentration rose from 295 p.p.m. in 1900 to approximately 390 p.p.m. in 2010 (refs 6, 7; see Matyssek and Sandermann^8^ for the possible effects of atmospheric composition on trees). This means an increase of more than 30% within nearly one century. Wet N-deposition increased by 0.5–1.0 kg ha^−1^ per decade^7^,^9^. Throughout Central Europe, average total N-deposition increased from approximately 2.5 kg ha^−1^ per year in 1900 to more than 9 kg ha^−1^ per year in the first decade of the twenty-first century^6^. Global average temperature has increased by roughly 0.7 °C (ref. 7) within the twentieth century. During the same period, the average temperature in Europe has risen by 0.95 °C (ref. 7). In Germany, the mean annual air temperature increased by 1.0 °C (ref. 10) during the twentieth century; and the sum annual precipitation increased by 9% during the same period. However, the annual distribution varied. During the winter months, precipitation increased by 19% during the last century, and rainfall in summer decreased by 3% on average. If only the second half of the twentieth century is measured, summer precipitation shows a 16% reduction.

In addition, the rise in atmospheric CO_2_ concentration, the higher N-deposition and the increase in air temperature were two to three times higher in the second half of the twentieth century compared with the first half. However, the strong temperature increase during the last 50 years reported for all of Europe^7^, but was not reported for Germany. The annual mean temperature increase for 1950–2000 was equal to the century average at 1.0 °C; only winter temperatures showed a higher increase in the second half of the twentieth century^10^. Higher temperatures will also extend the growing season^11^,^12^. Menzel and Fabian^11^ reported the average annual growing season has been extended by 10.8 days, since the early 1960s. Chmielewski and Rötzer^12^ also found the vegetation period was lengthened between 0.6 and 6.3 days per decade in different European natural regions during 1969–1998. Based on temperature data from the four climate stations used in this study (Supplementary Table 1), the length of the growing season, defined as the number of days annually with temperatures above 10 °C, was calculated for the last 110 years. Averaged over the climate stations, the growing season was extended by 22 days. The main increase, however, was detected over the last 50 years (Fig. 2).

This suggests notable wood volume growth rate increases at the stand level over the last 100 years (Fig. 1), coinciding with an increase in resource supply (CO_2_, N), together with an extended growing season accompanied by changes in other climatic variables (Fig. 2). These observations justified statistical analyses of growth trends, and model-based examination of the underlying mechanisms.

### Growth trends of key stand variables

**Figure 3:**
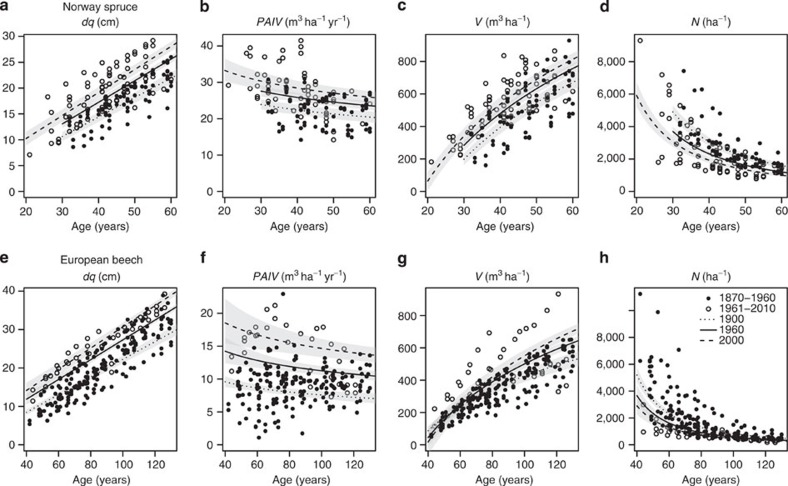
Statistical analysis of tree and stand dynamic changes since 1870. Trends in (**a**,**e**) mean stem diameter *dq*; (**b**,**f**) stand periodic annual volume increment (*PAIV*); (**c**,**g**) standing volume *V* and (**d**,**h**) tree number *N*; for Norway spruce (**a**–**d**) and European beech (**e**–**h**) age ranges. Observations before 1960 (filled symbols); after 1960 (empty symbols); predictions with our fitted linear mixed models (LMM) for 2000 (dashed line); for 1960 (solid line) and as a reference for 1900 (dotted line). The grey-shaded areas illustrate the prediction standard error. Although the error bands partly overlap, all illustrated calendar year trends were significant at *P*<0.05 (LMM), with *n*=157 (**a**,**c**,**d**); *n*=141 (**b**); *n*=225 (**e**,**g**,**h**) and *n*=217 (**f**). Note that positions on the error bands were not independent, for example, a prediction on the lower edge of the confidence band for one calendar year would be on the lower edge for all other calendar years.

**Figure 4:**
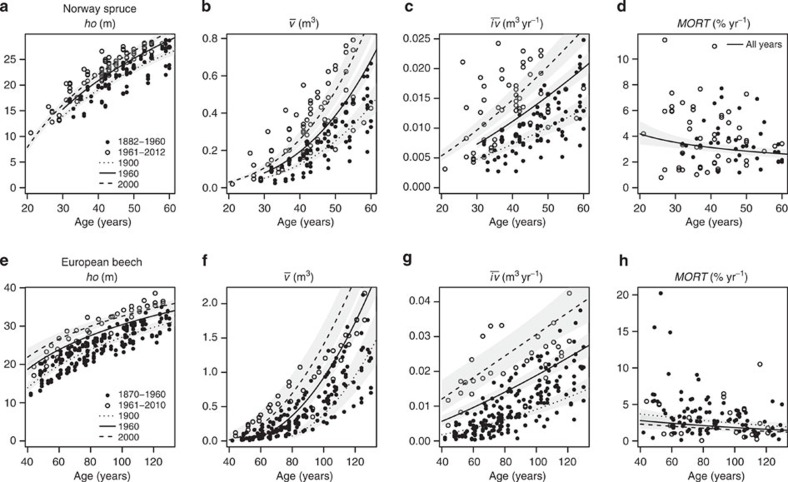
Additional statistical scrutiny of tree and stand dynamic changes since 1870. Trends in (**a**,**e**) dominant height *ho*; (**b**,**f**) mean tree volume 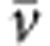; (**c**,**g**) mean tree annual volume increment 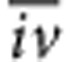; and (**d**,**h**) relative tree mortality rate, MORT; for Norway spruce (**a**–**d**) and European beech (**e**–**h**) age ranges. Observations before 1960 (filled symbols); after 1960 (empty symbols); predictions with our fitted linear mixed models (LMM) for 2000 (dashed line); for 1960 (solid line); and as a reference for 1900 (dotted line). The grey-shaded areas illustrate the prediction standard error. Although the error bands partially overlap, all illustrated calendar year trends are statistically significant at a minimum of *P*<0.05 (LMM), with *n*=157 (**a**,**b**); *n*=141 (**c**); *n*=225 (**e**,**f**); *n*=217 (**g**), with the exception of MORT in European beech (**h**) with *P*<0.1 (LMM, *n*=119) and no significance in Norway spruce (**d**). Note that positions on these error bands are not independent, for example, a prediction on the lower edge of the confidence band for one calendar year would be on the lower edge for all other calendar years.

First, we employed linear mixed models (LMMs) to determine whether the most important stand characteristics were dependent on only stand age, or also on calendar year. Standing wood volume (*V*), mean diameter (*dq*), dominant height (*ho*=mean height of the 100 tallest trees per hectare) and mean tree volume (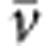) currently grow significantly faster than in the past (Figs 3, 4 and Supplementary Tables 2,3). Under the environmental conditions of the year 2000, any given mean diameter was attained following stand establishment up to more than one decade earlier than its counterpart in 1960 or before (Fig. 3). Stand volume growth, expressed as periodic annual increment of volume (PAIV) changed from 1960 to 2000 by, respectively, 10% and 30% for Norway spruce and European beech (Table 1). Most stands continued to accumulate volume, and have not reached a final constant yield plateau (Figs 1 and 3). For a 60-year-old Norway spruce stand, we expected a maximum standing volume *V* of 760 m^3^ ha^−1^ in 1960, and 810 m^3^ ha^−1^ in 2000. In a 130-year-old European beech stand, the expected maximum volume in 1960 was 630 m^3^ ha^−1^ compared with 700 m^3^ ha^−1^ in 2000 (Fig. 3). A consequence of accelerated stand development was a more rapid decrease in stand tree number *N* per unit area (Fig. 3), and change in tree mortality rate, MORT (Fig. 4). A comparison between 1960 and 2000 showed a 17% decrease in Norway spruce tree number and a 21% decrease in European beech. We did not detect a significant change in Norway spruce mortality rate, however, European beech exhibited a −17% change (Table 1). The calendar year effect on size and stand growth, and volume accumulation were significantly positive, and the calendar year effects on tree numbers were significantly negative. Significance levels obtained with LMM were at least *P*<0.05 with sample sizes *n*=157 (*V*, *dq*, *ho*, 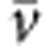, *N*) and *n*=141 (PAIV) for Norway spruce, and *n*=225 (*V*, *dq*, *ho*, 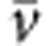, *N*) and *n*=217 (PAIV) for European beech (see Supplementary Tables 2, 3). In contrast, the negative calendar year effects on European beech mortality rates were only significant at the *P*<0.1 level (LMM, *n*=119), whereas there was even no significant effect for Norway spruce (Supplementary Table 3).

**Figure 5:**
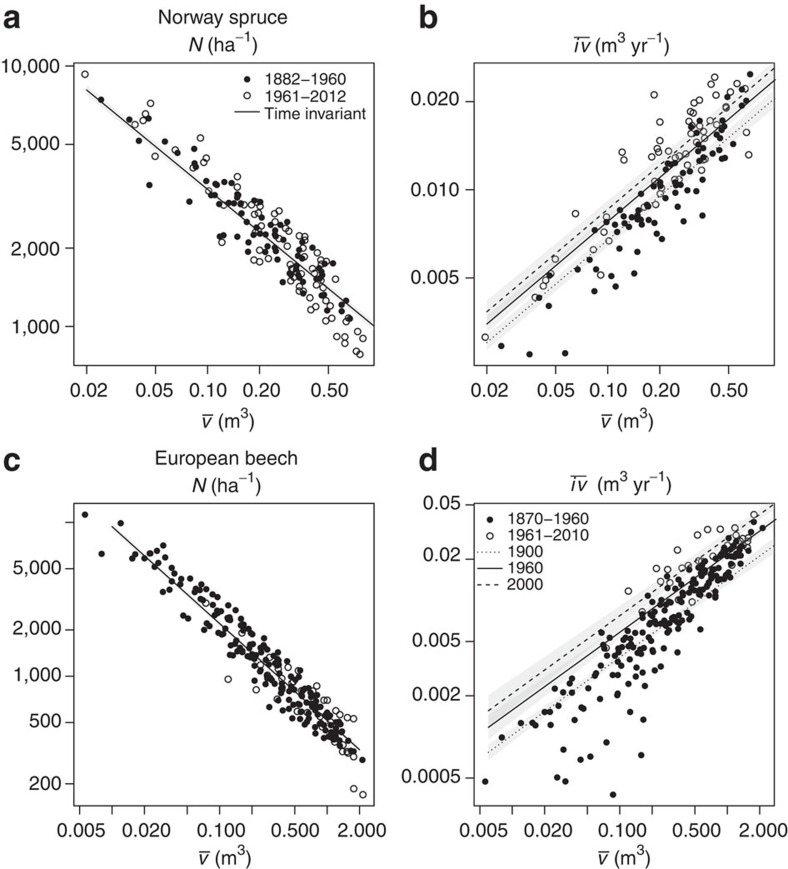
Stand allometry in past and present for Norway spruce and European beech. Relationships between (**a**,**c**) tree number *N* and mean tree volume 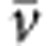; and (**b**,**d**) mean annual volume growth 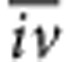 and mean tree volume 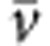 in a double-logarithmic scale for Norway spruce (**a**,**b**) and European beech (**c**,**d**). Filled symbols: observations up to 1960; empty symbols: after 1960. Predictions derived from our fitted linear mixed models (LMMs) do not change with calendar year for the 
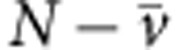
 relationship (solid lines in **a**,**c**), whereas they do for the 
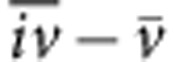
 relationship. We show the predictions for the years 2000 (dashed lines in **b**,**d**), 1960 (solid lines in **b**,**d**) and the 1900 reference (dotted lines in **b**,**d**). The grey-shaded areas illustrate the prediction standard error. Although the error bands partially overlap, all illustrated calendar year trends were significant at *P*<0.01 (LMM, *n*=141 and *n*=217 for Norway spruce and European beech). Note that positions on these error bands are not independent, for example, a prediction on the lower edge of the confidence band for one calendar year would be on the lower edge for all other calendar years.

We further tested whether stand allometry^2^,^13^, the relationship between average growth rate per tree and mean tree volume (
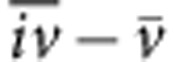
 relationship) and the relationship between tree number *N* and mean tree volume (
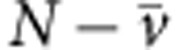
 relationship, self-thinning line) changed over time (Fig. 5). Results indicated self-thinning parameters did not significantly change with the calendar year (Fig. 5a,c and Supplementary Table 4). Regarding the 
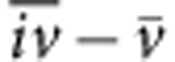
 relationship, the slope remained independent from the calendar year, however, the level significantly increased from past to present (Fig. 5b,d and Supplementary Table 4). Parameter estimates indicated the relative growth rate changed by 25% in Norway spruce, and 57% in European beech from 1960 to 2000 (Table 1, Supplementary Table 4. Both species showed similar slopes for the same allometric relationship (
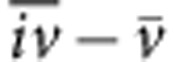
 relationship and 
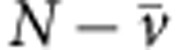
 relationship), as predicted by the Metabolic Scaling Theory^13^.

Therefore, present forest stands grow more rapidly, and accumulate a given standing volume earlier than comparable stands did a century ago. Regression results suggest the stands grow along a self-thinning line similar in slope and growth levels as in the past, but pass more rapidly through this usual 
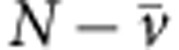
 trajectory. Consequently, the identified growth trend was primarily based on a changed relationship between tree size and growth. Remarkably, the change in the 
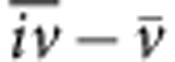
 relationship was manifested only in the curve’s level and not in its slope, the allometric coefficient. In Table 1, we used a 75-year reference age, which was the approximate harvest rotation age. We showed for stands of this age, stand characteristics changed from 1960 to 2000. Both years (1960 and 2000), as well as stand age (75 years), were inserted in the statistically fitted model functions to quantify the relative changes (equations 1, 2 and Supplementary Tables 2–4). Although tree height increased marginally, mean tree diameter and most notably mean tree volume increment showed accelerated change. Beech stand volume growth (+30%) and standing volume accumulation (+7%) proceeded significantly faster, so that tree number at a given age was already 21% lower than in the past. As the species’ self-thinning lines showed no significant upward shift during the whole time span covered by our data, decreased beech mortality rate (−17%) cannot be attributed to delayed mortality. Rather, it can be explained by the fact that in even-aged stands, mortality rate decreases continuously with stand age under steady-state conditions. Seemingly, owing to the more rapid growth, decreased mortality rates are reached significantly earlier than five or more decades ago. The fact that spruce and beech grew more rapidly, but stands still followed similar self-thinning became most obvious by 32–77% increased mean tree volume increments over time, an upward shift in growth-size allometry by 25–57%, and continued self-thinning. Our findings that self-thinning remained constant, while growth rates increased indicated the stock of resources have not changed, while the growth velocity and turnover have altered over time. Note, that changes in the reported order of magnitude are relevant for forest ecology and management.

### Site dependency of accelerated stand growth dynamics

**Figure 6:**
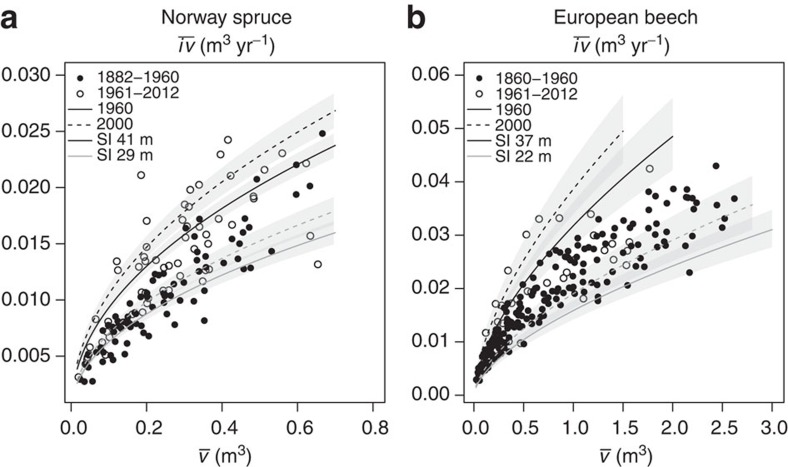
Site-dependent allometry change. The 
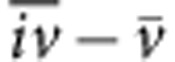
 relationship’s change (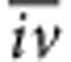: mean annual volume growth; 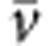: mean tree volume) depended on site quality as expressed by the site index (SI) for Norway spruce (**a**) and European beech (**b**). SI is the yield-table expectation for stand height at 100 years of age. Filled symbols: observations up to 1960; empty symbols: after 1960. Dashed lines represent the estimated curves obtained from fitted linear mixed models (LMM) for the calendar year 2000; solid lines for 1960. Light grey and black lines: curves for the lowest and highest SI represented by our data, respectively. The grey-shaded areas illustrate the prediction standard error. Although the error bands partly overlap, the illustrated interactions of calendar year and site quality were significant at *P*<0.01 (LMM, *n*=141 and *n*=217 for Norway spruce and European beech). Note that positions on these error bands were not independent, for example, a prediction on the lower edge of the confidence band for a given calendar year and a given site quality would be on the lower edge for all other calendar years and SI’s.

To arrive at a differential diagnosis to explain accelerated forest dynamics in Central Europe, we first statistically analysed if the change in 
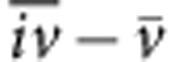
 relationships, clearly responsible for observed growth trends, were dependent on site quality. The latter was quantified using a site index (SI) based on actual stand height–age relationships and standard yield tables. This is common forestry practice, as the height a forest stand reaches at a given age is a reliable indicator of the growing conditions at a given location. The analysis revealed the slope depicting an increased trend in volume increments was caused by environmental change in both species, that is, the relative climate change benefits in terms of gains in stand productivity was significantly greater under better site conditions compared with poor sites (equation 3, Fig. 6 and Supplementary Table 5).

**Figure 7:**
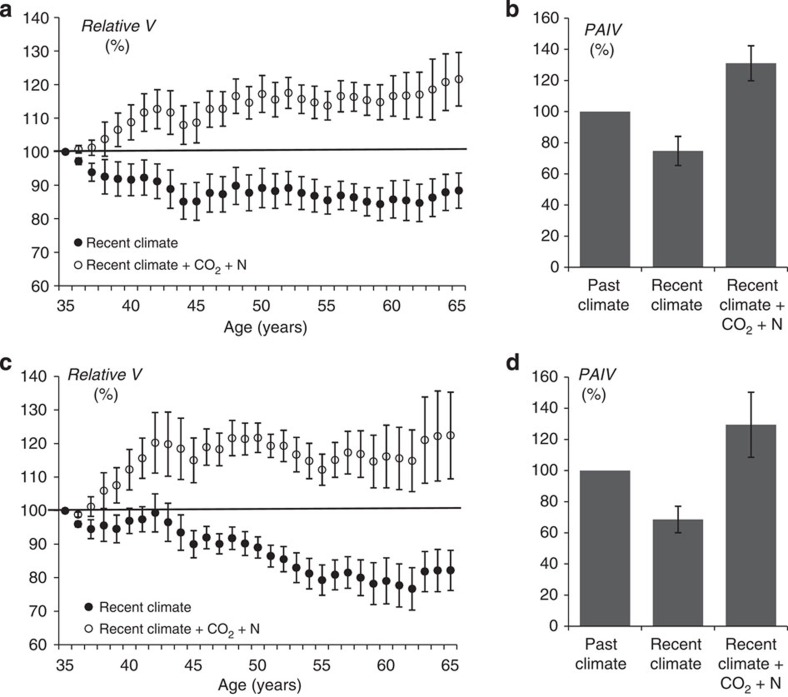
Scenario analysis based on the ecophysiological growth model BALANCE. Simulated relative standing volume (*V*; **a**,**c**) and average relative stand periodic annual volume increment (*PAIV*; **b**,**d**) with standard errors (*n*=4) for European beech (**a**,**b**) and Norway spruce (**c**,**d**) over 30 years for recent climatic conditions (1981–2010), recent climate with additional increased CO_2_ concentrations, and N deposition (100% base: past climate 1901–1930).

We further complemented our empirical studies with simulation experiments based on the ecophysiological model BALANCE^1^. We simulated forest growth under historical and recent environmental conditions, with the former as a reference. Simulations showed standing volume stock clearly decreased for scenarios where past climate conditions (1901–1930) were substituted by recent climate conditions (1981–2010); results were detected in both species, but most notably in Norway spruce (Fig. 7). Model results revealed climate change alone, that is, changes in temperature and precipitation, including an extended growing season, did not fully explain the observed growth trend in Norway spruce and European beech. However, if changes in air chemistry were considered in addition, the simulated stand volume and average annual stand volume increment in beech and spruce for recent Central European conditions exceeded the values for past environmental conditions, as empirically demonstrated. On fertile sites, the observed environmental change patterns resulted in increased growth acceleration, facilitating defined forest stand sizes, standing stock and developmental stages decades earlier than in the beginning of the twentieth century. In contrast, on sites with mineral nutrient limitations, environmental changes accelerated growth to a lesser extent. Thus, long-term survey data statistical analyses (Fig. 6 and Supplementary Table 5) and modelling scenario analyses (Fig. 7) suggested current increased temperatures and extended growing season contributed more to growth acceleration when site mineral nutrient supply was greater.

## Discussion

Studies in long-term ecosystem dynamics and change should consider past and present anthropogenic and natural causes. Long-term time series analyses on ecological processes and climate were applied to better understand ecosystem behaviour in the American Southwest^14^. Based on tree ring analysis, Swetnam *et al*. revealed an unprecedented ramp in tree growth since the mid-1970s, and attributed the observations to recovery from a 1950s extreme drought period, anomalous warming and mild wet winters associated with El Niño events^14^. The study did not completely rule out anthropogenic effects, such as CO_2_ enrichment.

The increased stand growth revealed in our study surprisingly occurred during the period when acid rain (1970–1990) and drought episodes (1976 and 2003) suggest decreased productivity should have occurred. One possible explanation for these results includes acid rain, after long-distance transport, only affected rather restricted areas of the Central European highland mountain tops (for example, Ore Mountains, Black Forest, Bavarian Forest or Bohemian Forest), but rarely the lowland forests, where the experimental areas used in this study are located^15^,^16^. The 1976 and 2003 droughts were the most severe in Europe’s recent climate history. However, both droughts were rather short lived, and an upward growth trend occurred immediately following each drought^17^. Longer drought periods, as expected under future climatic conditions^7^,^18^, might cause much longer lags in tree growth.

Kahle *et al*. provided evidence for growth trends at the tree level^19^, however, our approach showed the relevance of growth trends for stand level productivity. A broad scientific community was made aware of such trends in the 1990s^20^, however, to date, statistical inference beyond the case study level was missing. Our data, which covered an observational time span of more than one century, and even included records from subsequent stands at the same locations, revealed statistically significant growth changes from past to present. These unique time series analyses and results reflected how climate change actually modified the various components of forest stand dynamics. Tree and stand development are driven by resources rather than mere age^13^, and because of increased resource availability, both aged faster than in the past. As predicted by allometric theory^13^, the stands passed along continuous self-thinning lines^21^, which reflected site carrying capacity^22^. However, to date, because of increased growth rates, stands achieved defined sizes, standing stock and stand development stages significantly earlier than in the past. In other words, an average tree exhibited accelerated growth, but at any given average tree size, the maximum tree packing density per unit area did not change.

Our simulation results were consistent with biosphere model evaluations in response to climate variability, nitrogen and CO_2_ concentration changes^23^,^24^,^25^,^26^. Despite high variability in published models, overall results exhibit congruencies. The dependencies between carbon, water and nitrogen cycles as depicted in the model simulations are also obvious in empirical studies. For example, boreal Norway spruce growth was not increased due to higher CO_2_ concentrations, unless nutrients were supplied^27^. Further, FACE-experiments indicated that enhanced CO_2_ concentrations also affect nitrogen availability and plant water supply^25^,^28^, whereas the effect of elevated CO_2_ concentrations on forest stand growth is still unclear, especially under long-term conditions^29^,^30^,^31^,^32^. The enhanced forest growth within the last decades found empirically and by model simulations within this study is in line with other results reported in the literature^33^,^34^. Most likely, the observed climate change patterns including extended growing seasons^11^,^12^, combined with higher N-depositions caused this increased growth^33^,^34^. Other nutrient supplies in addition to N (ref. 35), extreme events^24^, soil conditions^36^, elevated ozone^37^, stand structure, that is, species composition^38^ and scaling to the landscape level^39^ can further modify the effect of higher temperature, and changed precipitation patterns on stand productivity.

The accelerated tree growth and forest ageing requires conformance of all associated organisms, including humans. Plants and animals inhabit these habitats, and depend on special phases in stand development and structure; faster growth means interference in species living conditions, and demands for higher mobility^40^. European beech profited more from changed growing conditions, therefore, Norway spruce might become a weaker competitor against beech and hence lose ground on the long run. In forestry practices, more rapid growth in size can result in earlier harvest threshold diameters and rotation because of increased stand productivity, which can raise the annual cut. Recent growth trends allow foresters to maintain much higher standing stocks. However, strong thinnings, which use earlier conditions as a guideline, might reduce stand density such that the actual growth potential is not fully realized. For a specific mean tree size, standing stock and mortality rate can be achieved one or more decades earlier, this leaves age-based experience values, widely used yield tables and other models, and many traditional management guidelines to become obsolete^1^. In addition, a shortened rotation period can mean reduced risk in terms of forest damage, including bark beetle infestation, windthrow and/or snow breakage.

Assuming growth acceleration is caused by higher resource supplies during a lengthened growing season, similar growth trends can be expected for a forest area of more than 45 × 10^6^ ha from Northern Germany to Slovenia, and France to Hungary^7^. Because our findings were based on continuously unthinned or at most moderately thinned forest stands, but always fully stocked experimental stands, progress in silvicultural practices can be excluded as cause for the observed growth trend. In the primarily intensively thinned stands from routine forest practices in Central Europe, the positive effects of thinning on tree and stand growth might even contribute to a climate change-induced growth trend. Tree breeding can also be excluded as a cause for observed growth trends, as on our research plots in many cases subsequent stands at the same location are of the same genotype. Other relevant species in this area, including Sessile oak (*Quercus petraea* L.) and Scots pine (*Pinus sylvestris* L.) dominate on less fertile sites than those typically stocked with Norway spruce and European beech, so that the benefit of the additional resource supply and growth acceleration might be even higher^41^. The increased growth rate, harvest and standing stock accumulation can be expected to heighten the carbon turnover rate in Central European forests. We roughly estimated the wood-related share in this rate by assuming an additional annual volume growth of 3 m^3^ ha^−1^ per year (~0.75 t C) on a 45 × 10^6^ ha area, which resulted in 34 × 10^6^ t C per year additionally stored in wood at first. However, whether this accelerated turnover translates into actual additional C sequestration by increasing forests’ standing stock or the stock of long-living wood products is highly questionable and cannot be answered with our data. Raising standing stocks may be undesired in managed forests as higher stand densities correlate with a higher risk of storm or snow damage. Intensified harvest, on the other hand, might result in new local problems because of mineral nutrient export comparable to former litter raking. Yet, it offers a chance for substituting fossil-based products in a C-neutral way, even if wood is only put into short-lived use like energy provision. Currently, it is unclear whether growth trends will remain positive under future climate change^7^. Detection, analysis and understanding can only be gained from continued long-term observation plots.

Hans Carl von Carlowitz (1645–1714) initiated long-term plots, and other founding forestry fathers followed by quantifying sustainable forestry practices at regional levels. Unfortunately, most plots were viewed as out-dated when it was time to inventory forests, considered too expensive and abandoned. However, our study emphasized the unique contribution of long-term observational plots to regional and global ecosystem monitoring, ecological research and environmental policy. Even 300 years after von Carlowitz proposed the concept of sustainable forests by publishing *Sylvicultura Oeconomica*^42^ in 1713, long-term plots remain the ultimate arbiters of human footprints on forest ecosystems.

## Methods

### Observational plots used in this study

Our empirical data were derived from 58 long-term observational plots in Germany; 36 supported Norway spruce and 22 European beech stands (Supplementary Tables 6–10). The plots cover a range of northern latitudes between 47.78° and 51.63°, and eastern longitudes between 7.92° and 13.31° (Supplementary Fig. 1). They belong to an extensive network of long-term research plots, which is scientifically maintained under the first author’s and his group’s responsibility (see Supplementary Note 1 for background information). All 58 plots selected for this study were either unthinned or moderately thinned, but always fully stocked. This focus on stands with no or minor silvicultural impact on stand dynamics, which were strictly and consistently maintained under scientific control, excluded treatment and treatment changes as reasons for the observed growth trends.

The unthinned (‘A-grade’^43^) experimental plots established in the nineteenth century possess considerable value and information potential for eco-monitoring. In Europe’s entirely managed forests, the unthinned plots represent an exceptional case of 140 years of unmanaged ecosystem development, where growth trends are not confounded by any silvicultural treatment effects. The potential for slight interference on A-grade plots because of forest protection occurs, however, if any, only dead and suppressed dying trees with negligible influence on stand dynamics are removed^43^. In addition, this study also used so-called B-grade plots, where the living basal area is slightly reduced, but without impact to the crown layer^43^. This is understood as a slight advance on natural mortality. A similar concept has been applied on the ‘80%-plots’ used in this study, where stand basal area is never reduced to less than 80% of an accompanying A-grade plot. Supplementary Table 10 provides a plot-wise list of thinning intensities. We exclusively selected stands established with high initial stand densities (tree number per hectare >5,000) to assure only fully stocked stands were included. All stands are monospecific and even-aged; they originated from planting or seeding. We used such long-term observational plots to determine whether growth rate changed over time, and how changes affected tree growth, standing volume, stand density, mortality and other stand attributes.

The selected plots represent growth conditions in the plains and highlands of Middle and Central Germany (Supplementary Fig. 1). The localities occur from 330 to 843 m above sea level. Although European beech plots dominate the Atlantic plains and highlands climate, Norway spruce plots are located in submontane and montane highlands, and the pre-alpine mountain zone (Supplementary Tables 6–8). The long-term mean temperature and annual precipitation exhibits a broad range across both species (5.7–8.5 °C and 605–1,369 mm per year), as well as for each species separately (for example, for Norway spruce, 6.9–8.3 °C and 812–1,256 mm per year). The plot distribution over 13 eco-regions and 11 geological zones is reflected by the broad spectrum of soil types. The poorest soils are podsols derived from sandstone and cretaceous material in the Palatinate and Upper Palatinate regions; the most fertile soils are parabrown soils from diluvial loess-loam in the pre-alpine highlands. The majority of stands are supported on soils of mediocre fertility on Triassic, Jurassic and Cretaceous formations between Frankfurt and Munich (Supplementary Fig. 1, see Supplementary Tables 6 and 8 for further details).

The data set comprises plots in present day mature stands surveyed up to 18 times since 1870, but also in young stands established in the last decade and only surveyed twice. Hence, the plots cover both historic and recent growth behaviour under respective environmental conditions. The broad variation of stand age (21–188 years), dominant height (10.7–44.4 m), tree number per hectare (133–11,238 trees ha^−1^) and quadratic mean tree diameter (5.4–54.4 cm) show the plots represent a rather wide range of stand developmental stages. Total yield (50−2,459 m^3^ ha^−1^), standing volume (50–1,637 m^3^ ha^−1^) and periodic annual volume increment (7.1–41.5 m^3^ ha^−1^ per year), as well as SI (19.8–43.1 m) emphasize the wide spectrum of site conditions and productivity levels (*cf.* Supplementary Table 9).

### Survey and evaluation of long-term observational plots

We based our analyses on the International Union of Forest Research Organisations^2^ standard variables, which quantified above ground mean tree and stand stem volume, rather than single tree volume or biomass. Therefore, additional assumptions for scaling from volume to mass were avoided; however, it required the following variable definitions: (i) all stand variables relate to a unit area of 1 ha (10^4^ m^2^); (ii) tree diameter, *dq* (cm), refers to the quadratic mean diameter at breast height (1.30 m) for all trees per plot; (iii) dominant height, *ho* (m) is the mean height of the 100 tallest trees per hectare; (iv) mean tree volume, 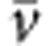 (m^3^) is the arithmetic mean stem volume; (v) annual tree volume growth, 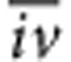 (m^3^ yr^−1^) is the mean annual volume growth of the mean trees with volume 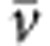; (vi) PAIV (m^3^ ha^−1^ per year) refers to the entire stand’s mean annual stem volume growth during a period between two surveys; (vii) standing stand volume, *V* (m^3^ ha^−1^) is the accumulated stem volume per hectare; (viii) the successive surveys of remaining, dead and harvested trees generates the current tree number per unit area, *N* (ha^−1^), and enables the calculation of the annual tree mortality rate, MORT (% per year); and (ix) the plots’ SI is expressed as measured or expected stand height at age 100 years. We used the prevalent yield tables by Assmann and Franz^4^, and Schober^5^ for Norway spruce and European beech, respectively. For complementary details, see Supplementary Methods.

### Dependency of stand variables on age and calendar year

We examined whether stand development on observational plots reflects any long-term growth trends by modelling stand characteristics dependent on stand age and calendar year. Certainly, stand characteristics from successive surveys (for example, PAIV, standing volume (*V*) and tree mortality rate (MORT) depend on age. An additional calendar year effect on stand characteristics would indicate a growth trend; if stands at a defined age perform differently in different calendar periods or decades, this will indicate a change in growth and site conditions. For investigating this, the following basic LMM structure was the most appropriate:


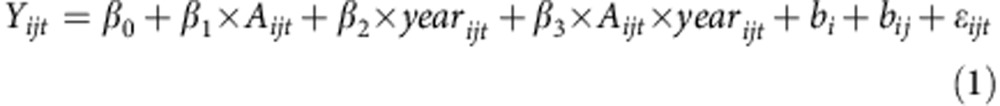


Variable *Y* represents the stand characteristic of interest (for example, PAIV, *V* and so on), untransformed or logarithmized, depending on whether the logarithmic transformation rendered a better model fit. Similarly, *A* represents stand age, untransformed or its logarithm. Choosing appropriate combinations of *Y* and *A* logarithmic and untransformed values allowed us to sufficiently cover nonlinear age-dependent relationships with a linear regression model. The second explanatory variable, calendar year, corresponding to a given observation, is indicated by the variable *year*.

The indices *i*, *j* and *t* represent the location an observational plot is included, the plot itself and the point of time a plot survey has occurred. Fixed effects parameters are *β*_0_–*β*_3_, whereas *b*_*i*_ and *b*_*ij*_ are location and plot random effects (*b*_*i*_~N(0,*τ*_1_^**2**^), *b*_*ij*_~N(0,*τ*_2_^**2**^)). Including these random effects, we avoid biased results due to the plot-specific and possibly also location-specific autocorrelation among the observations. Finally, *ε*_*ijt*_ denotes i.i.d. errors *(ε*_*ijt*_~N(0,*σ*^2^)).

The calendar year effect and its interaction with age (represented by *β*_2_ and *β*_3_ parameters) were only maintained in the model when they were statistically significant. Otherwise, the model was reduced accordingly and fitted again. If the interaction was significant, but not the isolated year effect, both were maintained in the model^44^.

High stand ages in our data primarily occurred with recent calendar years only, therefore, we excluded specific observations beyond a certain age to develop a balance of age-calendar year combination data set. Our models were fitted for 60 years and younger stand ages in Norway spruce, and 130 years and younger in European beech. For most stand characteristics as response variables this resulted in a sample size of *n*=157 and *n*=225 for Norway spruce and European beech, respectively. For the growth variables PAIV and 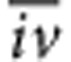 the sample size reduced to *n*=141 (spruce) and *n*=217 (beech) as there is no increment information available for the plots’ last surveys. The mortality rate, MORT, could be meaningfully analysed for the completely unthinned plots only which results in *n*=90 (spruce) and *n*=119 (beech). All models were fitted by maximizing the restricted maximum likelihood criterion (*cf.* Zuur *et al*.^44^).

### Allometric relationships of stand growth and size variables

The relationships between mean tree growth and mean tree size (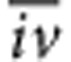 versus 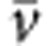), and tree number per unit area and mean size (*N* versus 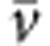) are cornerstones of allometric theory^45^,^46^,^47^,^48^,^49^. In the double logarithmic scale, both relationships follow a straight line ln(*y*)=*a*+*b* × ln(*x*) (equivalent to *y*=*e*^*a*^ × *x*^*b*^) with rather general and species-overarching values for the slope *b*. However, it is widely accepted that line levels, represented by intercept *a*, depend on environmental conditions and species^21^,^50^,^51^. We used a LMM, which is very similar to the basic model shown above to test the extent both allometric relationships are influenced by calendar-year-dependent trends:


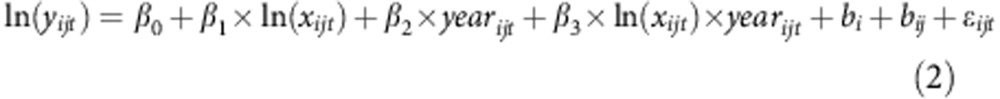


where *y* and *x* represent 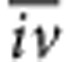 and 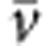, or *N* and 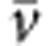, respectively. The variable and index names are defined the same as in equations 1. We employed exactly the same data used in fitting the age trend models, including only stands younger than 61 (Norway spruce) and 131 (European beech) years.

### Site dependency of the 
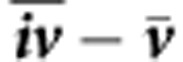
 relationship’s temporal trend

Results of the previous analyses suggested an upward shift with time (significant parameter *β*_2_ in equation 2) in the allometric relationship between 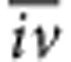 and 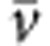 as the common mechanism underlying the observed growth trends. With the same data, we tested any change in allometry dependent on site conditions by formulating the following linear mixed regression model:


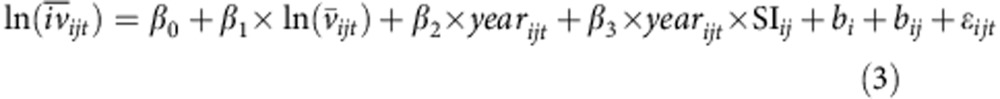


where SI is the respective plot’s SI, expressed as an expected stand height at an age of 100 years (see above). The other variable meanings and names are defined exactly the same as above. If parameter *β*_3_ differs significantly from zero, this indicates the allometric shift depends on site quality. All statistical analyses were performed with R 3.0.2 (ref. 52).

### Growth trend and changed arrival age at threshold values

To quantify how stand characteristics changed, we choose stand age 75 years, calculated how the stands perform at that age in 2000, and divided that by stand performance at the same age in 1960. For this purpose, both values were derived for the respective stand variables from the fitted model equations using the fixed effects parameter estimates (equations 1, 2 and Supplementary Tables 2–4) while setting the random effects to zero. For *PAIV*, for example, this procedure yields *PAIV*_age 75,2000_ and *PAIV*_age 75,1960_, and the ratio *RPAIV*_age 75,2000/1960_=*PAIV*_age 75,2000_/*PAIV*_age 75,1960_, which reflects the growth trend since 1960.

The age when a mean tree variable or a stand characteristic arrives at a defined threshold value is a practical and relevant measure. Assume the fixed effects parameters of equations 1 have been estimated, we have the following equation for estimating 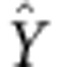, which is the general expected value for a given stand characteristic or its logarithm:


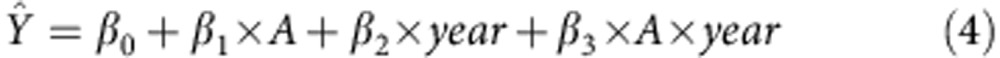


This can be rearranged as


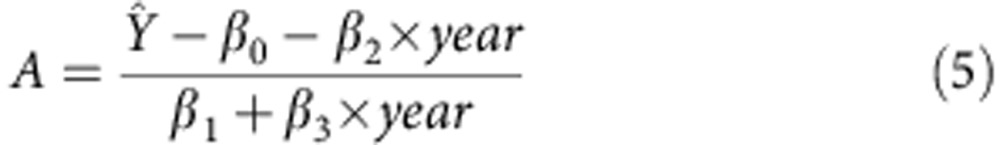


which allows us to estimate *A*, the mean age (or its logarithm) when a certain threshold value 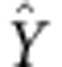 is reached under the environmental conditions of a given year.

### Process-based modelling

The physiological growth model BALANCE^53^,^54^ we used for scenario analyses simulates the three-dimensional development of individual trees in a stand, and estimates the consequences of environmental influences. Tree development is calculated as a response to individual environmental conditions, and as environmental conditions change with individual tree development, the influences of competition, stand structure, species mixture and management options can be assessed with the model (Supplementary Fig. 2).

Initial tree biomass is calculated from the dimensional variables tree height, height to crown base, diameter at breast height, tree position and crown radii. Biomass increase is the result of the interaction between physiological processes, which are dependent on the physical and chemical microenvironment. These are in turn influenced by stand spatial structure. Asymmetric crown shapes are included, and generate a spatially explicit representation of the environment. The calculation levels vary from stand level to individual trees, from tree components (that is, foliage, branches, stems, and fine and coarse roots) to crown and root layers, which are spatially subdivided into segments. Consequently, an increase in biomass is simulated based on the carbon and nitrogen uptake from each segment, depending on its energy supply and resource availability.

By using weather data, microclimate and water balance are simulated for each layer and segment, respectively. Air temperature and radiation within the stand is calculated for every crown layer of every tree on the basis of leaf area distribution for the respective tree and its competitors. The spatial distribution of light and water availability is estimated on a daily basis. Water balance simulation examines soil conditions in different soil layers, where vertical and horizontal water flows between rooted and non-rooted fractions are considered. Based on the Penman-Monteith^55^ approach, potential evapotranspiration is estimated, from which the actual evapotranspiration of a tree is calculated using maximum water uptake derived from water content within soil volume pervaded by fine roots. Water can be exchanged between rooted and un-rooted soil layers. Total soil water content is reduced by drainage, which is equivalent to percolation from the deepest soil layers.

Foliage biomass and leaf area as well as light availability and photosynthetically active radiation (PAR) absorption change with the onset of bud burst. A tree’s foliage emergence date determines its assimilation and respiration rate, but also alters the environmental conditions in the immediate surrounding area. Bud burst of a tree species is estimated using an air temperature sum model, whereas foliage senescence is simulated depending on the respiration sum for each segment of a tree. Based on the aggregated driving variables, all physiological processes, that is, assimilation, respiration, nutrient uptake, growth, senescence and allocation can be calculated for each individual tree. Nutrient uptake is the result of demand, supply and absorption capacity, whereby demand is based on the difference between the actual nitrogen concentration, and a given optimal concentration. Supply is defined by soil characteristics of the rooted volume, uptake capacity by root surface, and its specific absorption rate.

Physiological processes are calculated in 10-day time steps using aggregated results of daily environmental conditions. Gross primary production is estimated depending on leaf surface, absorbed PAR, temperature, CO_2_ concentration, water and nitrogen supply (Supplementary Fig. 3).

Total respiration is the sum of maintenance losses and growth respiration. Maintenance respiration is calculated for each segment as a function of biomass, specific respiration rate and temperature. Growth respiration is estimated as a constant fraction of maximum photosynthesis. The fixed carbon not required for respiration is distributed into plant compartments, including foliage, branches, stems and roots. The available carbon for allocation is apportioned into different compartments according to growth and respiration demands. Carbon allocation is defined by the relationships between the compartments according to the functional carbon balance theory^56^, and the pipe model theory^57^. Consequently, all tissues within a segment, that is, foliage and branches, or fine and coarse roots, as well as the amount of stem wood, are mechanistically linked to each other. Dimensional tree growth is estimated annually, based on biomass accumulation during that year. Volume expansion depends on the necessary amount of twigs and transport branches, and the amount of coarse roots within root segments. Therefore, crown development is preferred in the direction of best assimilation conditions during the previous year. If net assimilation rates are negative, the crown segment is regarded as dead. If no segments contain living biomass, the tree is assumed dead and removed from calculations.

BALANCE has been extensively validated for basic micro-meteorological and physiological processes, for water balance, annual tree development and entire stand development^54^,^58^. Detailed descriptions of BALANCE, and single modules can be obtained from Grote and Pretzsch^53^, or Rötzer *et al*.^54^

### Scenarios calculated with BALANCE

Climate data from four German climate stations with daily time series for more than 100 years formed the foundation of the growth simulations using BALANCE. Supplementary Table 1 shows the geographical coordinates, mean air temperature and precipitation values for the chosen simulation periods. These four stations are representative of most climate regions in Central Europe. We chose a sandy loam soil type with an available field capacity of 186 mm to a maximum rooting depth of 1 m. Field capacity decreases from 35 to 25 vol% with increasing soil depth, whereas the wilting point was set constant at 8 vol%. Plant available N was assumed low at the beginning of the simulation. This was justified because historically, forests in Germany were displaced by agriculture to nutrient poor sites.

Growth development of a 30-year-old Norway spruce and 35-year-old European beech stand (Supplementary Table 11) was simulated for time spans 1901–1930 and 1981–2001. The first simulation scenario (reference) was the 1901–1930 period using the daily climate record obtained from each station, and continuously increasing atmospheric CO_2_ concentrations from 295 p.p.m. to 307.p.p.m., and N-depositions from 6 kg N ha^−1^ per year to 7 kg N ha^−1^ per year. The second scenario reproduced the recent climate conditions from 1981 to 2010 while keeping N-depositions and CO_2_ concentration on the previous level. In scenario 3, an increase of atmospheric CO_2_ concentrations from 338 p.p.m. from 1981 to 389 p.p.m. in 2010, and increased N-deposition from 15 kg N ha^−1^ per year in 1981 to 20 kg N ha^−1^ per year in 2010 was included in the model. Scenario 3 thus integrates all recent environmental conditions. Atmospheric CO_2_ concentration and N-deposition data were derived from Churkina *et al*.^6^ In this way, the single and overall influences of climate, CO_2_ and N-deposition were analysed.

## Author contributions

H.P. initiated the study, interpreted the data and wrote the paper. P.B. performed statistical analyses, interpreted the data and wrote the paper. G.S. compiled the data. E.U. interpreted the data and revised the manuscript. T.R. performed and interpreted simulation runs and wrote the paper.

## Additional information

**How to cite this article**: Pretzsch, H. *et al*. Forest stand growth dynamics in Central Europe have accelerated since 1870. *Nat. Commun.* 5:4967 doi: 10.1038/ncomms5967 (2014).

## Supplementary Material

Supplementary Information Supplementary Figures 1-3, Supplementary Tables 1-11, Supplementary Notes 1, Supplementary Methods and Supplementary References.

## Figures and Tables

**Table 1:** Change of the characteristics of 75-year-old forest stands 2000 in relation to 1960.

**Forest stand attribute**	**Change from 1960–2000 in %**	
	**N. spruce**	**E. beech**
Dominant tree height, *ho*	**+6**	**+9**
Mean tree diameter, *dq*	**+9**	**+14**
Mean tree volume, 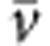	**+34**	**+20**
Stand volume growth, PAIV	**+10**	**+30**
Standing volume stock, *V*	**+6**	**+7**
Tree number, *N*	**−17**	**−21**
Mortality rate, MORT	NS	−17
Mean tree volume		
increment 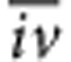	**+32**	**+77**
Shift of 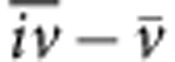 -allometry	**+25**	**+57**
Shift of 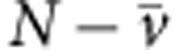 -allometry	NS	NS

E. beech, European beech; N. spruce, Norway spruce; *PAIV*, periodic annual increment of volume.

Comparative changes between 2000 and 1960 determined from our fitted linear mixed models (LMMs). We only report changes based on significant calendar year effects; bold numbers: *P*<0.05 (LMM); normal number: *P*<0.10 (LMM). Sample sizes for Norway spruce: *n*=157 (*ho, dq*, 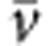
*, V, N*, 
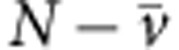
-allometry), *n*=141 (PAIV, 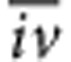, 
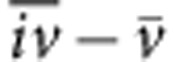
-allometry), *n*=90 (MORT). Sample sizes for European beech: *n*=225 (*ho, dq*, 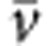
*, V, N*, 
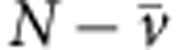
-allometry), *n*=217 (PAIV, 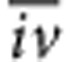, 
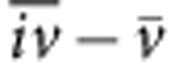
-allometry), *n*=119 (MORT). The crucial calendar year effects for a given forest stand attribute might result from one or two significant parameter estimates.
