# Supplementary material for: Forest stand growth dynamics in Central Europe have accelerated since 1870
Source: Nat Commun. 2014 Sep 12;5:4967. doi: 10.1038/ncomms5967 (PMC4175583; doi:10.1038/ncomms5967)
Supplement: Supplementary Information — Supplementary Figures 1-3, Supplementary Tables 1-11, Supplementary Notes 1, Supplementary Methods and Supplementary References. [file ncomms5967-s1.pdf]

Supplementary Figure 1 | Geographic position of the long-term observational locations included in this study

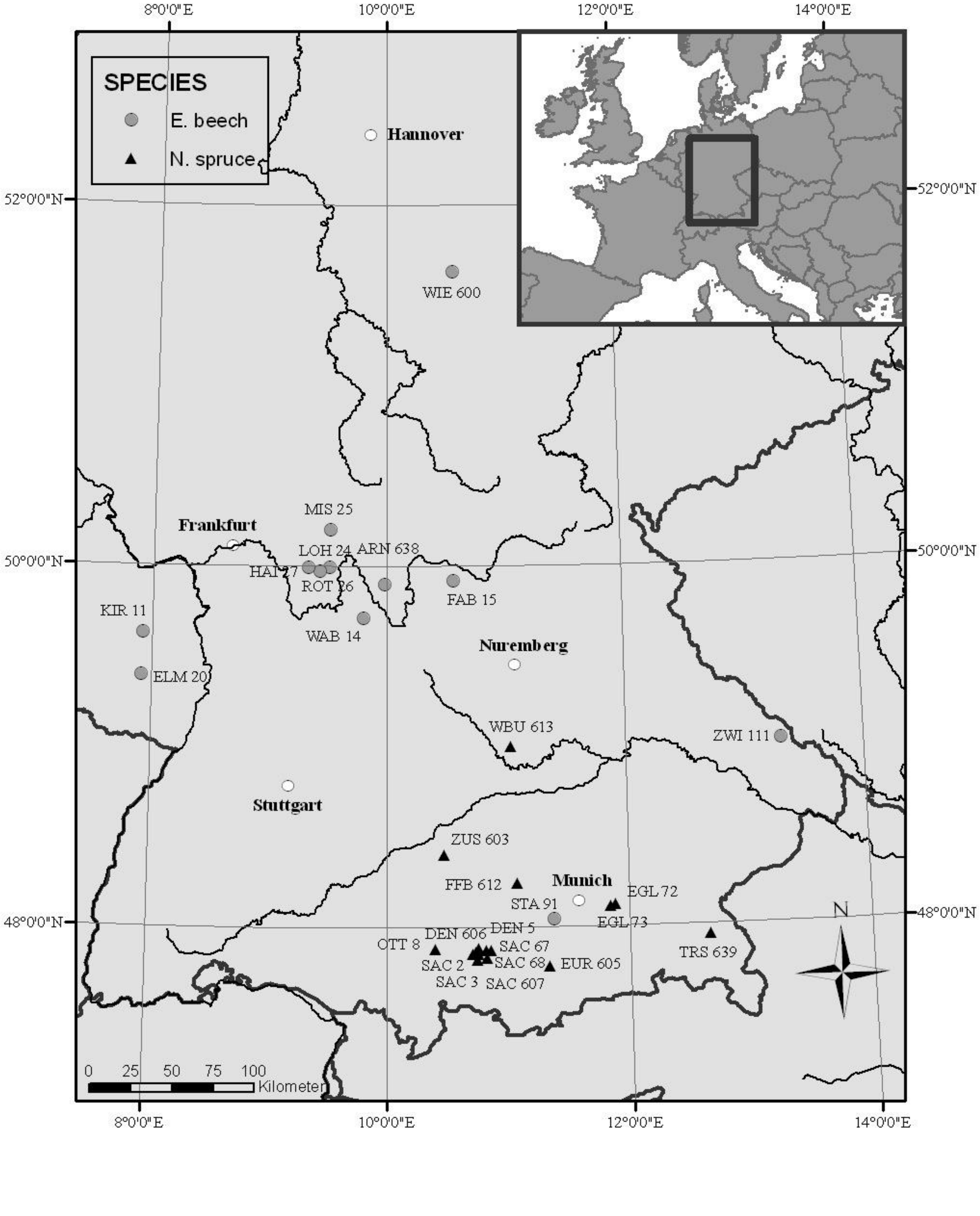

Locations are represented by species specific symbols (● European beech, *Fagus sylvatica* L.), ▲ Norway spruce, (*Picea abies* (L.) Karst.), by abbreviation, and by location number. One location might comprise several plots.

Supplementary Figure 2 | Functional diagram of the eco-physiological growth model BALANCE

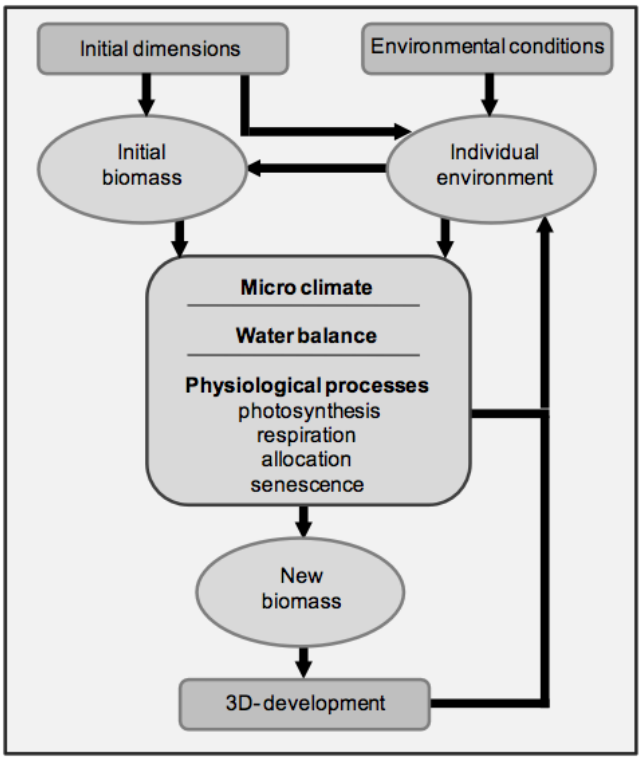

Supplementary Figure 3 | Basic physiological relationships as implemented in the eco-physiological growth model BALANCE

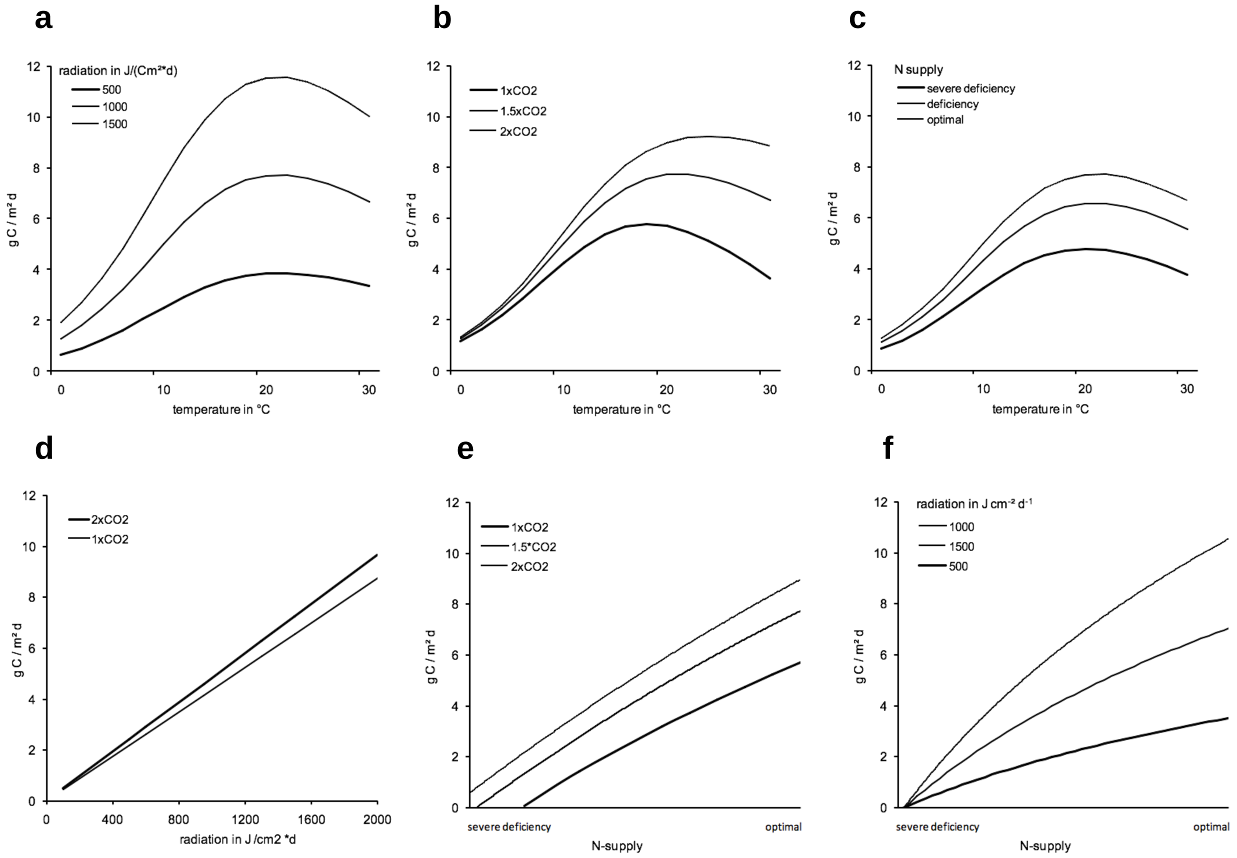

Dependency of gross primary production (*GPP*) from temperature and radiation (a), temperature and CO_2 concentration (b), temperature and N-supply (c), radiation and CO_2 concentration (d), N-supply and CO_2 concentration (e); and N-supply and radiation (f); (1 x CO_2 = current atmospheric CO_2 concentration; 1.5 x CO_2 and 2 x CO_2 = increase in CO_2 concentration by 50% and 100%)

Supplementary Table 1 | Location and climate parameters of the four climate stations used in this study (data: Deutscher Wetterdienst[1])

**climate station**	longitude [°]	latitude [°]	altitude [m asl]	temperature [°C] 1901-1930	temperature [°C] 1981-2010	precipitation [mm yr^{-1}] 1901-1930	precipitation [mm yr^{-1}] 1981-2010
Hamburg	9.983	53.633	11	8.7	9.4	752	792
Hohenpeissenberg	11.017	47.800	977	6.2	7.3	1084	1175
Karlsruhe	8.367	49.033	112	10.1	11.1	767	784
Potsdam	13.067	52.383	81	8.5	9.3	590	585

Coordinates, altitude, mean air temperature and precipitation values for the two simulation periods (Karlsruhe data were available up to the year 2007 only).

Supplementary Table 2 | Results of the mixed model regressions with quadratic mean diameter, periodic annual volume increment, standing stand volume, and tree number per hectare as goal variables

			Fixed Effects				Random Effects		
Species	Response Variable Y	Age Variable A	β_0	β_1	β_2	β_3	τ_1^2	τ_2^2	σ^2
N. spruce	*dq*	*age*	-38.0817<br>*0.2055*	-0.9370 **<br>*0.0020*	0.0197<br>*0.2106*	0.0007 ***<br>*0.0000*	6.7652	3.1181	0.4068
	ln(*PAIV*)	ln(*age*)	-0.5687<br>*0.7347*	-0.2269 ***<br>*0.0002*	0.0024 *<br>*0.0108*	n.s.	0.0336	0.0009	0.0102
	V	ln(*age*)	-4822.235 ***<br>*0.0000*	682.3650 ***<br>*0.0000*	1.4209 *<br>*0.0147*	n.s.	14357.07	392.9184	1304.73
	ln(*N*)	ln(*age*)	22.9208 ***<br>*0.0000*	-1.6217 ***<br>*0.0000*	-0.0047 ***<br>*0.0003*	n.s.	0.0365	0.0550	0.0146
E. beech	*dq*	*age*	-74.1330 *<br>*0.0404*	-0.6409 *<br>*0.0271*	0.0386 *<br>*0.0456*	0.0005 **<br>*0.0023*	0.9829	6.9606	2.1562
	ln(*PAIV*)	ln(*age*)	-9.3436 ***<br>*0.0000*	-0.2562 *<br>*0.0496*	0.0066 ***<br>*0.0000*	n.s.	0.0224	0.0038	0.1210
	V	ln(*age*)	14929.19 ***<br>*0.0000*	-3665.29 ***<br>*0.0000*	-8.5295 ***<br>*0.0000*	2.1237 ***<br>0.0000	7024.14	2383.86	1553.43
	ln(*N*)	ln(*age*)	27.0359 ***<br>*0.0000*	-2.0054 ***<br>*0.0000*	-0.0058 **<br>*0.0022*	n.s.	0.0000	0.1582	0.0375

Quadratic mean diameter *dq*, periodic annual volume increment *PAIV*, standing stand volume *V*, and tree number per hectare *N* were response variables of linear mixed model regressions (LMM) dependent from stand age and calendar year. The model equation was $Y_{ijt} = \beta_0 + \beta_1 \times A_{ijt} + \beta_2 \times year_{ijt} + \beta_3 \times A_{ijt} \times year_{ijt} + b_i + b_{ij} + \varepsilon_{ijt}$ with the indices *i*, *j*, *t* representing location, plot in location, and survey point of time. The model contains the random effects $b_i \sim N(0,\tau_1^2)$, $b_{ij} \sim N(0,\tau_2^2)$ and the i.i.d. errors $\varepsilon_{ijt} \sim N(0,\sigma^2)$. Significance levels: * $P < 0.05$, ** $P < 0.01$, *** $P < 0.001$. Exact *P*-values are given in italics below the parameter estimates. The number of observations was 157 (141 for *PAIV*) and 225 (217 for *PAIV*) for Norway spruce and European beech, respectively.

Supplementary Table 3 | Results of the mixed model regressions with dominant height, mean tree volume, mean tree volume increment, and relative tree mortality rate as goal variables

			Fixed Effects				Random Effects		
Species	Response Variable *Y*	Age Variable *A*	β_0	β_1	β_2	β_3	τ_1^2	τ_2^2	σ^2
N. spruce	ho	ln(*age*)	85.9584 *<br>*0.0127*	-33.6287 ***<br>*0.0001*	-0.0698 ***<br>*0.0001*	0.0271 ***<br>*0.0000*	3.5918	0.4101	0.1916
	ln($\bar{v}$)	ln(*age*)	-27.3773 ***<br>*0.0000*	3.1083 ***<br>*0.0000*	0.0073 ***<br>*0.0004*	n.s.	0.1489	0.0508	0.0178
	ln($\overline{iv}$)	ln(*age*)	-23.6619 ***<br>*0.0000*	1.4602 ***<br>*0.0000*	0.0070 ***<br>*0.0000*	n.s.	0.0775	0.0574	0.0241
	ln(*MORT*)	ln(*age*)	2.6917 **<br>*0.0069*	-0.4210<br>*0.1061*	n.s.	n.s.	0.1036	0.0339	0.2067
E. beech	*ho*	ln(*age*)	-381.8653 ***<br>*0.0000*	66.1673 ***<br>*0.0000*	0.1804 ***<br>*0.0002*	-0.0273 **<br>*0.0016*	4.6953	0.3214	0.9461
	$\bar{v}^2$	*age*	-11.8699 ***<br>*0.0004*	0.0177 ***<br>*0.0000*	0.0056 **<br>*0.0017*	n.s.	0.0247	0.0307	0.2339
	ln($\overline{iv}$)	ln(*age*)	-100.5495 ***<br>*0.0003*	15.7892 *<br>*0.0111*	0.0462 **<br>*0.0013*	-0.0074 *<br>*0.0226*	0.0000	0.1853	0.1471
	ln(*MORT*)	*age*	10.5660 *<br>*0.0264*	-0.0070 •<br>*0.0811*	-0.0047 •<br>*0.0635*	n.s.	0.0101	0.0000	0.7495

Dominant height *ho*, mean tree volume $\bar{v}$, mean tree volume increment $\overline{iv}$, and relative tree mortality rate *MORT* were response variables of linear mixed model regressions (LMM) dependent from stand age and calendar year. The model equation was $Y_{ijt} = \beta_0 + \beta_1 \times A_{ijt} + \beta_2 \times year_{ijt} + \beta_3 \times A_{ijt} \times year_{ijt} + b_i + b_{ij} + \varepsilon_{ijt}$ with the indices *i*, *j*, *t* representing location, plot in location, and survey point of time. The model contains the random effects b_i~N(0,τ_1^2), b_{ij}~N(0,τ_2^2) and the i.i.d. errors ε_{ijt}~N(0,σ^2). Significance levels: • $P < 0.10$, * $P < 0.05$, ** $P < 0.01$, *** $P < 0.001$. Exact *P*-values are printed in italics below the parameter estimates. The number of observations was 157 (141 for $\overline{iv}$, 90 for *MORT*) and 225 (217 for $\overline{iv}$, 119 for *MORT*) for Norway spruce and European beech, respectively. Unthinned plot observations were used for *MORT* as a single goal variable.

Supplementary Table 4 | Results of the mixed model regressions for fundamental allometric relationships and their dependency on the calendar year

			Fixed Effects				Random Effects		
Species	Response Variable y	Size Variable x	β_0	β_1	β_2	β_3	τ_1^2	τ_2^2	σ^2
N. spruce	N	$\bar{v}$	6.8647 *** 0.0000	-0.5454 *** 0.0000	n.s.	n.s.	0.0137	0.0121	0.0064
	$\overline{iv}$	$\bar{v}$	-8.4405 *** 0.0000	0.5004 *** 0.0000	0.0024 ** 0.0056	n.s.	0.0149	0.0170	0.0153
E. beech	N	$\bar{v}$	6.2453 *** 0.0000	-0.6301 *** 0.0000	n.s.	n.s.	0.0000	0.0400	0.0132
	$\overline{iv}$	$\bar{v}$	-17.5598 *** 0.0000	0.5697 *** 0.0000	0.0070 *** 0.0000	n.s.	0.0000	0.0402	0.1364

The allometric relationships of number of trees per hectare N (response variable y) *versus* mean tree volume $\bar{v}$ (size variable x), and mean tree volume increment $\overline{iv}$ (response variable y) versus mean tree volume $\bar{v}$ (size variable x) were tested for dependency on calendar year *year* with a linear mixed model (LMM).
The model equation was $\ln(y_{ijt}) = \beta_0 + \beta_1 \times \ln(x_{ijt}) + \beta_2 \times year_{ijt} + \beta_3 \times \ln(x_{ijt}) \times year_{ijt} + b_i + b_{ij} + \varepsilon_{ijt}$ with the indices i, j, t representing location, plot in location, and survey point of time. The model contains the random effects $b_i \sim N(0, \tau_1^2)$, $b_{ij} \sim N(0, \tau_2^2)$ and the i.i.d. errors $\varepsilon_{ijt} \sim N(0, \sigma^2)$. Significance levels: * $P < 0.05$, ** $P < 0.01$, *** $P < 0.001$. Exact P-values are printed in italics below the parameter estimates. The number of observations was 157 (141 for $\overline{iv}$) and 225 (217 for $\overline{iv}$) for Norway spruce and European beech, respectively.

Supplementary Table 5 | Results of the mixed model regressions for calendar year and site dependent allometric shift

			Fixed Effects				Random Effects		
Species	Response Variable	Size Variable	β_0	β_1	β_2	β_3	τ_1^2	τ_2^2	σ^2
N. spruce	$\overline{iv}$	$\bar{v}$	-9.5642 *** *0.0000*	0.4810 *** *0.0000*	0.0024 ** *0.0028*	0.000017 *** *0.0000*	0.0168	0.0045	0.0153
E. beech	$\overline{iv}$	$\bar{v}$	-13.0522 *** *0.0000*	0.6109 *** *0.0000*	0.0040 * *0.0141*	0.000024 ** *0.0018*	0.0000	0.0220	0.1370

The dependency of the allometric relationship between mean tree volume increment $\overline{iv}$ and mean tree volume $\bar{v}$ from calendar year *year* and site index *SI* (expected stand height at age = 100 years, according to standard yield tables[2,3]) was examined with a linear mixed regression model (LMM) according to the equation $\ln(\overline{iv}_{ijt}) = \beta_0 + \beta_1 \times \ln(\bar{v}_{ijt}) + \beta_2 \times year_{ijt} + \beta_3 \times year_{ijt} \times SI_{ij} + b_i + b_{ij} + \varepsilon_{ijt}$ with the indices *i*, *j*, *t* representing location, plot in location, and survey point of time. The model contains the random effects $b_i \sim N(0,\tau_1^2)$, $b_{ij} \sim N(0,\tau_2^2)$ and the i.i.d. errors $\varepsilon_{ijt} \sim N(0,\sigma^2)$. Significance levels: * $P < 0.05$, ** $P < 0.01$, *** $P < 0.001$. Exact *P*-values are printed in italics below the parameter estimates. The number of observations was 141 and 217 for Norway spruce and European beech, respectively.

Supplementary Table 6 | Site characteristics for the 58 observational plots in Norway spruce (*n* = 36) and European beech (*n* = 22) included in this analysis

Observational plot: location/ plot number(s)	Species	Stand age at last survey (years)	Geographic position E-longitude	N-latitude	Elevation a. s. l. [m]	Mean annual temp. [°C]	Mean annual precipitation [mm]	Soil conditions soil moisture, substrate
Sachsenried 2/1, 2	N. spruce	122	10.75	47.85	820	7.0	1,149	moderatly moist, deep silt
Sachsenried 3/1, 2		116	10.76	47.85	830	7.0	1,131	moderatly moist, deep silt
Denklingen 5/1, 2		143	10.84	47.97	782	7.3	1,067	moderatly moist, deep silt
Ottobeuren 8/1, 2		113	10.40	47.88	660	6.9	1,322	moderatly moist, deep silt
Sachsenried 67/1,2		131	10.75	47.83	843	6.9	1,220	moderatly moist, deep silt
Sachsenried 68/1,2		130	10.75	47.83	843	6.9	1,223	moist, deep silt
Eglharting 72/1, 2		120	11.85	48.12	533	8.3	1,044	moderatly moist, sandy silt
Eglharting 73/1, 2		119	11.85	48.11	541	8.3	1,071	moderatly moist, sandy silt
Denklingen 84/2		127	10.83	47.87	781	7.3	1,097	moist, deep silt
Sachsenried 602/1		46	10.76	47.85	820	7.0	1,131	moist, silty coarse clay
Zusmarshausen 603/1, 2, 3		47	10.48	48.40	510	8.2	863	moderatly moist, sandy silt
Eurach 605/7, 8		55	11.34	47.78	600	8.0	1,304	moist, silt
Denklingen 606/3, 4		55	10.83	47.87	750	7.2	1,126	moist, deep silt
Sachsenried 607/3, 7, 8, 9, 10		53	10.82	47.87	775	7.2	1,124	moderatly moist, silty clay
Fürstenfeldbruck 612/7, 19		43	11.08	48.24	542	8.1	932	moderatly moist, silt
Weißenburg 613/2, 4, 7		93	11.04	49.00	560	7.6	812	moist, silt
Traunstein 639/1		41	12.67	47.94	590	8.1	1,356	moderatly moist, sandy silt
* Kirchheimbolanden 11/1, 2	E. beech	114	7.92	49.62	640	8.0	690	dry, silty sand
Waldbrunn 14/1, 2		145	11.19	49.71	360	7.8	892	moderatly moist, silt
Fabrikschleichach 15/1, 2		188	10.57	49.92	460	7.7	700	moist, sand
* Elmstein 20/1,2		145	7.92	49.39	500	8.0	850	dry, silty sand
Lohr 24/1, 2		162	9.51	49.99	500	7.9	959	moist, sand
Mittelsinn 25/1, 2		170	9.52	50.20	510	8.2	860	moist, sand
Rothenbuch 26/1, 2		144	9.43	49.97	450	7.7	1,010	moderatly moist, sand
Hain 27/1, 2		172	9.33	49.99	400	8.0	889	moderatly moist, sand
Starnberg 91/2, 4		78	11.38	48.04	620	8.0	1,054	moderatly dry, sandy silt
Zwiesel 111/2, 4		126	13.31	49.07	760	5.7	1,369	moderatly moist, sandy-stony silt
* Wieda 600/2		121	10.58	51.63	360	7.0	1,100	moist, silt
Arnstein 638/1		65	9.98	49.90	330	8.5	605	moist, silty coarse clay

The table indicates stand age, geographic position, altitude a.s.l., mean annual temperature, annual precipitation, and soil conditions for each plot at the last survey. For most plots, climate data were derived from current climate maps based on mean values from 1971 to 2000[4]. If marked with '*', climate values were provided by the state forest service of Rhineland-Palatinate.

Supplementary Table 7 | Assignment of the 58 observational plots of Norway spruce and European beech stands to ecoregions and sub-ecoregions in Germany

Observational Plot: Location/plot number(s)	Species	Ecoregion	Sub-Ecoregion
Sachsenried 2/1, 2	N. spruce	Schwäbisch-Bayerische Jungmoräne und Molassevorberge	Oberbayerische Jungmoräne und Molassevorberge
Sachsenried 3/1, 2		Schwäbisch-Bayerische Jungmoräne und Molassevorberge	Oberbayerische Jungmoräne und Molassevorberge
Denklingen 5/1, 2		Schwäbisch-Bayerische Jungmoräne und Molassevorberge	Oberbayerische Jungmoräne und Molassevorberge
Ottobeuren 8/1, 2		Schwäbisch-Bayerische Schotterplatten und Altmoränenlandschaft	Vorallgäu
Sachsenried 67/1,2		Schwäbisch-Bayerische Jungmoräne und Molassevorberge	Oberbayerische Jungmoräne und Molassevorberge
Sachsenried 68/1,2		Schwäbisch-Bayerische Jungmoräne und Molassevorberge	Oberbayerische Jungmoräne und Molassevorberge
Eglharting 72/1, 2		Schwäbisch-Bayerische Schotterplatten und Altmoränenlandschaft	Isentaler Altmoräne und Hochterrasse
Eglharting 73/1, 2		Schwäbisch-Bayerische Schotterplatten und Altmoränenlandschaft	Isentaler Altmoräne und Hochterrasse
Denklingen 84/2		Schwäbisch-Bayerische Jungmoräne und Molassevorberge	Oberbayerische Jungmoräne und Molassevorberge
Sachsenried 602/1		Schwäbisch-Bayerische Jungmoräne und Molassevorberge	Oberbayerische Jungmoräne und Molassevorberge
Zusmarshausen 603/1, 2, 3		Tertiäres Hügelland	Mittelschwäbisches Schotterriedel- und Hügelland
Eurach 605/7, 8		Schwäbisch-Bayerische Jungmoräne und Molassevorberge	Oberbayerische Jungmoräne und Molassevorberge
Denklingen 606/3, 4		Schwäbisch-Bayerische Jungmoräne und Molassevorberge	Oberbayerische Jungmoräne und Molassevorberge
Sachsenried 607/3, 7, 8, 9, 10		Schwäbisch-Bayerische Jungmoräne und Molassevorberge	Oberbayerische Jungmoräne und Molassevorberge
Fürstenfeldbruck 612/7, 19		Schwäbisch-Bayerische Schotterplatten und Altmoränenlandschaft	Landsberger Altmoräne
Weißenburg 613/2, 4, 7		Frankenalb und Oberpfälzer Jura	Südliche Frankenalb und Südlicher Oberpfälzer Jura
Traunstein 639/1		Schwäbisch-Bayerische Jungmoräne und Molassevorberge	Oberbayerische Jungmoräne und Molassevorberge
Kirchheimbolanden 11/1, 2	E. beech	Saar-Nahe Bergland	Nordpfälzer Bergland
Waldbrunn 14/1, 2		Fränkische Platte	Südliche Fränkische Platte
Fabrikschleichach 15/1, 2		Fränkischer Keuper und Albvorland	Steigerwald
Elmstein 20/1,2		Pfälzerwald	Mittlerer Pfälzerwald
Lohr 24/1, 2		Spessart-Odenwald	Buntsandsteinspessart
Mittelsinn 25/1, 2		Spessart-Odenwald	Buntsandsteinspessart
Rothenbuch 26/1, 2		Spessart-Odenwald	Buntsandsteinspessart
Hain 27/1, 2		Spessart-Odenwald	Buntsandsteinspessart
Starnberg 91/2, 4		Schwäbisch-Bayerische Jungmoräne und Molassevorberge	Oberbayerische Jungmoräne und Molassevorberge
Zwiesel 111/2, 4		Bayerischer Wald	Innerer Bayerischer Wald
Wieda 600/2		Harz	Montaner Mittel- und Hochharz
Arnstein 638/1		Fränkische Platte	Südliche Fränkische Platte

For details regarding the given ecoregions see Arbeitskreis Standortskartierung[5].

Supplementary Table 8 | Condensed information about the observational plots used in this study

Characteristics	Norway spruce min	Norway spruce max	European beech min	European beech max
Number of plots (*n*)	36		22	
Eastern-longitude	10.40	12.67	7.92	13.31
Northern-latidute	47.78	49.00	48.04	51.63
Altitude a. s. l. (m)	510	843	330	760
Mean annual temperature (°C)	6.9	8.3	5.7	8.5
Annual precipitation (mm yr^{-1})	812	1356	605	1369

Overview of the number of plots, their geographic range (min-max), altitude a.s.l., mean annual temperature, and annual precipitation.

Supplementary Table 9 | Condensed overview of important observational plot stand characteristics

Characteristics	Norway spruce		European beech	
	min	max	min	max
Number of plots (*n*)	36		22	
First survey (year)	1882	2001	1870	1991
Last survey (year)	1963	2012	1936	2012
Number of surveys (*n*)	3	18	4	19
Age at last survey (yrs)	41	143	65	188
ho (m) last survey	24.3	44.4	28.7	40.7
N (ha^{-1}) la. Surv	344	2,229	133	924
SI (m) la. surv.	28.7	42.8	23.5	37.6
dq (cm) la. surv.	19.1	54.4	21.6	54.0
V (m^{3}ha^{-1}) la. surv.	563	1,637	328	1,119
PAIV (m^{3}ha^{-1}yr^{-1}) la. surv.	10.1	39.8	7.2	21.2
TY (m^{3}ha$^{-1)}$ la. surv.	682	2,459	531	1,565

Range of important stand characteristics for plots used in this study at the last. Dominant height (mean height of the 100 tallest trees per ha), *ho*; tree number, *N*; site index, *SI* (expected stand height at age = 100 years, according to standard yield tables[2,3]); quadratic mean diameter, *dq*; standing volume, *V*; periodic annual volume increment, *PAIV*; total yield from stand establishment to last survey, including removal stand, *TY*.

Supplementary Table 10 | Overview of growth and yield characteristics of the 58 observational plots included in this analysis

Observational Plot: Location/plot number(s)	Species	Thinning intensity	Stand age at last survey (years)	First survey (year)	Last survey (year)	Number of surveys	Dominant height *ho* (m)	Site index (m)	Tree number *N* (ha^{-1})	Quadratic mean diameter d_q (cm)	Standing volume *V* (m^3ha^{-1})	Periodic annual increment *PAIV* ($m^3ha^{-1}y^{-1}$)	Total yield *TY* (m^3ha^{-1})
Sachsenried 2/1	N. spruce	A	122	1882	1972	15	40.9	36.4	492	44.7	1,364	15.6	2,188
Sachsenried 2/2		B	122	1882	1972	15	42.4	39.8	372	49.1	1,247	14.0	2,297
Sachsenried 3/1		A	116	1882	1965	14	40.2	36.3	596	42.0	1,452	17.8	1,966
Sachsenried 3/2		B	116	1882	1965	14	40.2	38.3	480	45.2	1,338	15.9	1,953
Denklingen 5/1		A	143	1882	1990	18	42.0	36.5	496	47.6	1,505	16.5	2,147
Denklingen 5/2		B	143	1882	1990	18	42.8	38.9	388	54.0	1,597	17.0	2,288
Ottobeuren 8/1		A	113	1882	1963	14	40.8	37.2	632	40.6	1,473	14.5	2,216
Ottobeuren 8/2		B	119	1882	1969	15	42.0	39.7	476	45.3	1,425	14.0	2,312
Sachsenried 67/1		A	131	1902	1990	14	44.4	38.3	443	50.7	1,637	18.5	2,378
Sachsenried 67/2		B	131	1902	1990	14	44.1	40.7	344	54.4	1,453	18.2	2,459
Sachsenried 68/1		A	130	1902	1990	14	43.6	38.4	544	45.3	1,566	17.8	2,273
Sachsenried 68/2		B	130	1902	1990	14	43.2	40.0	376	50.3	1,365	16.3	2,301
Eglharting 72/1		A	120	1906	1990	14	34.7	28.7	600	37.5	912	10.1	1,487
Eglharting 72/2		B	120	1906	1990	13	38.0	35.8	556	41.0	1,200	18.3	1,736
Eglharting 73/1		A	119	1906	1983	12	35.5	31.1	672	36.9	1,123	16.4	1,489
Eglharting 73/2		B	119	1906	1983	12	36.4	34.3	520	39.9	1,064	17.8	1,605
Denklingen 84/2		B	127	1921	2007	13	39.7	36.9	472	46.8	1,331	14.9	2,166
Sachsenried 602/1		A	46	1989	2008	5	25.4	38.1	1,919	22.2	828	39.8	1,036
Zusmarshausen 603/1		A	47	1995	2010	4	26.9	38.0	2,214	20.0	809	31.6	975
Zusmarshausen 603/2		A	47	1984	2010	6	27.5	38.4	2,229	19.1	757	32.5	994
Zusmarshausen 603/3		A	47	1995	2010	4	27.0	39.4	1,735	22.1	791	27.8	872
Eurach 605/7		A	55	1997	2007	3	28.1	36.1	956	28.2	706	16.4	867
Eurach 605/8		A	55	1991	2007	4	28.4	36.1	900	29.2	713	14.2	873
Denklingen 606/3		A	55	1982	2008	6	27.9	35.6	1,778	22.8	865	27.3	1,159
Denklingen 606/4		A	55	1982	2008	6	26.5	34.7	1,800	22.2	814	25.3	1,058
Sachsenried 607/3		A	53	1982	2006	6	26.2	33.4	1,668	21.8	675	24.7	858
Sachsenried 607/7		80%	53	1994	2006	4	28.2	40.9	803	28.0	583	21.1	817
Sachsenried 607/8		80%	53	1994	2006	4	28.0	40.6	782	28.4	595	19.1	901
Sachsenried 607/9		80%	53	1994	2006	4	27.1	39.4	912	26.1	568	20.2	816
Sachsenried 607/10		A	53	1982	2006	6	27.3	35.1	1,076	24.1	563	21.6	793
Fürstenfeldbruck 612/7		A	43	2001	2012	3	24.3	38.5	1,409	23.7	657	30.6	682
Fürstenfeldbruck 612/19		A	43	1996	2012	4	25.4	39.6	1,502	22.9	681	30.3	727
Weißenburg 613/2		A	93	1974	2009	6	35.3	33.6	673	35.3	966	14.7	1,335

Weißenburg 613/4		A	83	1995	2009	3	32.0	32.1	667	30.7	665	12.8	1,138
Weißenburg 613/7		A	83	1976	2009	6	34.5	33.8	778	33.1	943	16.6	1,390
Traunstein 639/1		A	41	1995	2010	4	26.6	42.8	1,520	24.9	836	38.8	889
Kirchheimbolanden 11/1	E. beech	A	114	1871	1936	10	29.2	24.3	755	27.8	609	11.2	798
Kirchheimbolanden 11/2		B	114	1871	1936	10	31.5	27.8	438	34.0	603	10.1	906
Waldbrunn 14/1		A	145	1870	1967	15	31.1	23.5	650	29.3	638	11.6	947
Waldbrunn 14/2		B	145	1870	1967	15	34.2	27.6	383	36.5	678	12.5	1,060
Fabrikschleichach 15/1		A	188	1870	2010	18	37.7	27.9	381	44.1	1,119	8.3	1,460
Fabrikschleichach 15/2		B	188	1870	2010	18	40.3	31.1	180	54.0	875	12.8	1,565
Elmstein 20/1		A	145	1871	1967	13	36.8	29.0	400	36.0	735	11.9	991
Elmstein 20/2		B	145	1871	1967	13	36.5	29.3	303	35.5	538	9.7	811
Lohr 24/1		A	162	1871	1967	13	34.2	25.6	292	38.6	569	11.4	1,038
Lohr 24/2		B	162	1871	1967	13	34.8	27.5	203	43.9	536	10.8	1,047
Mittelsinn 25/1		A	170	1870	1998	17	33.2	26.0	189	39.4	397	8.3	1,138
Mittelsinn 25/2		B	182	1870	2010	19	31.0	23.7	133	44.0	328	7.2	977
Rothenbuch 26/1		A	144	1871	1967	14	35.8	28.2	425	37.0	796	10.8	1,046
Rothenbuch 26/2		B	144	1871	1967	14	36.0	29.2	303	39.9	678	11.1	1,067
Hain 27/1		A	172	1881	2004	17	37.9	29.3	272	45.8	881	10.0	1,268
Hain 27/2		B	172	1881	2004	17	40.7	32.1	164	53.4	784	10.6	1,425
Starnberg 91/2		A	78	1971	2005	6	32.3	35.7	865	25.7	693	21.2	824
Starnberg 91/4		80%	85	1971	2012	7	31.8	33.8	505	29.5	543	18.2	771
Zwiesel 111/2		80%	126	1954	2002	8	36.0	28.8	170	39.3	366	7.9	733
Zwiesel 111/4		A	126	1954	2002	8	36.5	28.7	300	35.7	529	9.1	770
Wieda 600/2		A	121	1953	2006	10	38.6	31.9	530	34.8	934	14.6	1,186
Arnstein 638/1		A	65	1991	2004	4	28.7	37.6	924	21.6	449	16.7	531

Plot-wise overview of important stand characteristics recorded from each plot’s last survey. For the last survey, the table presents the following essential stand characteristics: Dominant height (mean height of the 100 tallest trees per ha), *ho*; site index (expected stand height at age = 100 years, according to standard yield tables[2,3]), tree number, *N*; quadratic mean diameter, *dq*; standing volume, *V*; periodic annual volume increment, *PAIV*; total yield from stand establishment to last survey, including removal stand, *TY*. Plot thinning intensity ‘A’: unthinned; ‘B’: moderately thinned; ‘80%’: maintaining stand basal area 20% lower compared to ‘A’.

Supplementary Table 11 | Stand characteristics of the initial stands used for scenario runs with the eco-physiological growth model BALANCE

Stand	Age (approx.) [years]	Tree number [ha^{-1}]	Mean height [m]	Mean diameter [cm]	Wood Volume [m^3 ha^{-1}]
Norway spruce	30	3,615	11.8	10.6	260
European beech	35	4,738	11.3	7.6	138

Supplementary Note 1 | Long-term observational plots

The concept of forest sustainability was proposed by von Carlowitz[6] 300 years ago, and Hartig[7,8], Paulsen[9], Cotta[10], and Pfeil[11] introduced principles to establish this idea in practice. Consequently, farsighted researchers initiated establishment of long-term observational plots in the late 19th century to procure growth and yield data as a quantitative basis for sustainable forest management (see von Ganghofer[12], Verein Deutscher Forstlicher Versuchsanstalten[13]). The appropriate thinning type, severity, and intensity was a pivotal point for sustainable management, therefore most of the early experiments comprised plots in pure and mixed stands with different thinning grades, as well as unthinned reference plots. Many of the observational plots established in the 1870s have been re-measured approximately 20 times to date, and are still an essential component of the forest observation network in Central Europe. Based on the long-term survey data, fundamental forest growth and yield relationships for theory development[14], yield tables[2,3,15,16,17], and other decision support models[18,19], as well as thinning, spacing, and species-mixing recommendations for silviculture[20,21] have been developed. The founding fathers planned the experimental plots for the long-term, and established experiments intended to last 100-200 years throughout a stand’s lifetime.

Supplementary Methods | Complementary information about stand variable calculations

Standard evaluation of the observational plots

Stand characteristics were evaluated following DESER-Norm 1993[22]. Older plots, which were under continuous survey for more than one hundred years delivered rather unique information about the total volume yield (*TY*). Repeated surveys at the stand level were performed in several year intervals, and generated periodic annual volume increment (*PAIV*) values, i.e. mean annual growth rates over longer time intervals. Between two surveys at times t_1 and t_2, given wood volumes V_1 and V_2 of the remaining stand at t_1 and t_2, and $V_{removed}$, the volume which was removed (or died) in-between the surveys, *PAIV* is

$$PAIV = \frac{V_2 - V_1 + V_{removed}}{t_2 - t_1}$$ Supplementary Equation 1

The total volume yield *TY* at a given time t is obtained by integration of *PAIV* from the first observation t_0 up to t:

$$TY_t = \int_{t_0}^{t} PAIV\, dt$$ Supplementary Equation 2

Standing volume *V* at time *t* is derived from

$$V_t = \int_{t_0}^{t} PAIV\, dt - \int_{t_0}^{t} V_{removed\ dt}$$ Supplementary Equation 3

All volume information given in this study refers to merchantable wood volume (log diameter > 7 cm at the smaller end), including bark.

Relative tree mortality rate

The annual mortality rate during the interval between two plot observations was estimated using the formula for compound interest effect

$$C_2 = C_1 \times \left[1 + \frac{p}{100}\right]^n$$ Supplementary Equation4

with capital C_2 and C_1 at the end or beginning of an observation interval, respectively; annual interest rate p in percent; and interval length in years, n. For estimating the annual percentage of the tree mortality rate, it can be written as

$$N_2 = N_1 \times \left[1 + \frac{MORT}{100}\right]^n$$ Supplementary Equation 5

Where N_2 and N_1 are tree number at the respective end and beginning of an observation interval; *MORT* represents the relative annual tree mortality rate and n depicts the interval length in years. Solving and rearranging the formula with respect to *MORT* yields

$$MORT = \left(\sqrt[n]{\frac{N_2}{N_1}} - 1\right) \times 100.$$ Supplementary Equation 6

Supplementary References

1. Deutscher Wetterdienst. http://www.dwd.de/bvbw/appmanager/bvbw/dwdwww Desktop?_nfpb=true&_pageLabel=dwdwww_result_page&portletMasterPortlet_i1gsbDocumentPath=Navigation%2FOeffentlichkeit%2FKlima__Umwelt%2FKlimadaten%2Fkldaten__kostenfrei%2Fausgabe__tageswerte__node.html%3F__nnn%3Dtrue; last ref. Feb. 2013. (2013)
2. Assmann, E. & Franz, F. Vorläufige Fichten-Ertragstafel für Bayern. *Forstw Cbl* **84(1)**, 13-43 (1965).
3. Schober, R. Buchen-Ertragstafel für mäßige und starke Durchforstung, In: Schober, R. (1972) Die Rotbuche. Schr Forstl Fak Univ Göttingen u Niedersächs Forstl Versuchsanst 43/44, JD Sauerländer's Verlag, Frankfurt am Main, p. 333 (1967).
4. Hera, U., Rötzer, T., Zimmermann, L., Schulz, C., Maier, H., Weber, H., Kölling, C. Klima en détail -- Neue hochaufgelöste Klimakarten zur klimatischen Regionalisierung Bayerns. LWF aktuell **86**, 34-37 (2011).
5. Arbeitskreis Standortskartierung. Forstliche Wuchsgebiete und Wuchsbezirke in der Bundesrepublik Deutschland, Landwirtschaftsverlag GmbH, Münster-Hiltrup, p. 170 (1985).
6. von Carlowitz, H.C. Sylvicultura Oeconomica oder Haußwirthliche Nachricht und Naturmäßige Anweisung zur wilden Baum-Zucht, JF Braun, Leipzig, p. 430 (1713).
7. Hartig, G.L. Anweisung zur Holzzucht für Förster. Neue Akademische Buchhandlung, Marburg (1791).
8. Hartig, G.L. Anweisung zu Taxation der Forsten oder zur Bestimmung des Holzertrages der Wälder. Heyer Verlag, Gießen, p. 166 (1795).
9. Paulsen, J.C. Kurze praktische Anleitung zum Forstwesen. Verfaßt von einem Forstmanne. Detmold, p. 152 (1795).
10. von Cotta, H. Anweisung zum Waldbau. Arnoldische Buchhandlung, Dresden, Leipzig (1828).
11. Pfeil, W. Die deutsche Holzzucht. Verlag Baumgartner, Leipzig, p. 551 (1860).
12. von Ganghofer, A. Das Forstliche Versuchswesen, Augsburg, p. 505 (1881).
13. Verein Deutscher Forstlicher Versuchsanstalten. Beratungen der vom Vereine Deutscher Forstlicher Versuchsanstalten eingesetzten Kommission zur Feststellung des neuen Arbeitsplanes für Durchforstungs- und Lichtungsversuche. *AFJZ* **78**, 180-184 (1902).
14. Assmann, E. The principles of forest yield study. Pergamon Press, Oxford, New York, p. 506 (1970).
15. Jüttner, O. Eichenertragstafeln. In: Schober, R. (1971) Ertragstafeln der wichtigsten Baumarten. JD Sauerländer's Verlag, Frankfurt am Main, 12-25 and 134-138 (1955).
16. Schober, R. Ertragstafeln wichtiger Baumarten. JD Sauerländer's Verlag, Frankfurt am Main (1975).

17. Wiedemann, E. Kiefern-Ertragstafel für mäßige Durchforstung, starke Durchforstung und Lichtung, In: Wiedemann, E. (1948) Die Kiefer 1948. Verlag M & H Schaper, Hannover, p. 337 (1943).

18. Pretzsch, H., Biber, P., Ďurský, J. The single tree based stand simulator SILVA. Construction, application and evaluation. *Forest Eco Manage* **162**, 3-21 (2002).

19. Gadow, von K. Forsteinrichtung – Adaptive Steuerung und Mehrpfadprinzip. Universitätsdrucke Göttingen: p. 163 (2006).

20. Pretzsch., H. *et al.* Comparison between the productivity of pure and mixed stands of Norway spruce and European beech along an ecological gradient, *Annals of Forest Science* **67**, 712-723 (2010)

21. Pretzsch, H. *et al.* Productivity of mixed versus pure stands of oak (*Quercus petraea* (Matt.) Liebl. and *Quercus robur* L.) and European beech (*Fagus sylvatica* L.) along an ecological gradient. *Eur J For Res* **132**, 263-280 (2013).

22. Johann, K. DESER-Norm 1993. Normen der Sektion Ertragskunde im Deutschen Verband Forstlicher Forschungsanstalten zur Aufbereitung von waldwachstumskundlichen Dauerversuchen. Proc Dt Verb Forstl Forschungsanst, Sek Ertragskd, in Unterreichenbach-Kapfenhardt, 96-104 (1993).
